# Transcriptomic analysis of rice cultivars with distinct resistance mechanisms to *Xanthomonas oryzae* pv. *oryzicola* reveals novel components and candidate genes associated with bacterial leaf streak

**DOI:** 10.3389/fpls.2025.1613802

**Published:** 2025-09-09

**Authors:** Moe Moe Kyi Win, Wanchana Aesomnuk, Reajina Dumhai, Siriphat Ruengphayak, Julie E. Gray, Ido Bar, Vinitchan Ruanjaichon, Samart Wanchana, Siwaret Arikit

**Affiliations:** ^1^ Program of Agricultural Sciences, Faculty of Agriculture at Kamphaeng Saen, Kasetsart University, Nakhon Pathom, Thailand; ^2^ Rice Science Center, Kasetsart University, Nakhon Pathom, Thailand; ^3^ Institute for Sustainable Food, School of Biosciences, University of Sheffield, Sheffield, United Kingdom; ^4^ Centre for Planetary Health and Food Security, School of Environment and Science, Griffith University, Nathan, QLD, Australia; ^5^ National Center for Genetic Engineering and Biotechnology (BIOTEC), Pathum Thani, Thailand; ^6^ Department of Agronomy, Faculty of Agriculture at Kamphaeng Saen, Kasetsart University, Nakhon Pathom, Thailand

**Keywords:** BLS, RNA-seq, *Xoc*, DEGs, rice, defense response

## Abstract

*Xanthomonas oryzae* pv. *oryzicola* causes bacterial leaf streak, a rice disease that can lead to substantial yield losses. Although an *xa5*-based signaling pathway is known to confer resistance to this disease, alternative stable and broad-spectrum resistance mechanisms will be critical for sustainable management. In this regard, we characterized NDCMP49, a Thai rice variety with strong resistance to bacterial leaf streak but lacking *xa5*-based resistance. Transcriptomes of NDCMP49 were compared with DV85, a rice variety with *xa5*-based resistance, and HCS, a bacterial leaf streak susceptible variety, at 0- and 9-hours post-inoculation with *X. oryzae* pv. *oryzicola*. Analysis of differentially expressed genes revealed a less transcriptional response in the two resistant varieties than in the susceptible variety. Nonetheless, during the first nine hours of infection, all varieties showed differential expression of receptor-like kinases, NB-LRR proteins, WRKY and NAC transcription factors, heat shock proteins, and chitinases, indicating the involvement of pathogen pattern-triggered immunity and effector-triggered immunity pathways. Interestingly, genes previously associated with the *Xa21*-mediated resistance to closely related pathogen *Xanthomonas oryzae* pv. *oryzae* were also identified. The genotype-phenotype association analysis performed on 249 rice accessions showed that *RIR1b* has some InDels in the gene’s coding region, which separates the accessions according to response to *X. oryzae* pv. *oryzicola*. Moreover, 2 bp insertions in the regulatory region led to up-regulation of *RIR1b* in NDCMP49 over DV85 and HCS. The genes identified here are valuable candidates for functional characterization. Targeting these genes would advance breeding rice varieties with strong resistance to bacterial leaf streak and other major diseases.

## 1 Introduction

Rice cultivation is important in ensuring food security and sustaining the livelihoods of millions around the world (FAO, 2023). Despite its vital role as a staple food crop, production faces significant threats from a multitude of diseases that impact both its yield and quality. Among these, bacterial leaf blight (BLB), bacterial leaf streak (BLS), sheath blight, rice blast, and various viral infections pose considerable challenges to rice cultivation (Liu et al., 2021). Of particular concern to our study is BLS, which ranks as one of the foremost bacterial diseases affecting rice; under conditions conducive to bacterial proliferation, it has the potential to cause yield losses of up to 32% (He et al., 2012). BLS is caused by the biotrophic, gram-negative bacterium *Xanthomonas oryzae* pv. *oryzicola* (*Xoc*), which not only affects plant health but also poses economic risks to farmers (Ke et al., 2017; Niño-Liu et al., 2006). The prevalence and distribution of BLS are primarily confined to tropical and subtropical regions, with notable incidences reported in several Asian countries. Moreover, its geographical range extends into rice-growing areas in Australia and West Africa, underscoring the extensive threat that BLS presents to rice production in diverse agricultural landscapes (Niño-Liu et al., 2006).

*Xoc* enters rice leaves through stomata and wounds, where it proliferates within the sub-stomatal cavities before ultimately colonizing the mesophyll parenchyma cells. The resultant symptoms are water-soaked, translucent to yellow lesions that appear as long, narrow, linear streaks between the veins along the leaf surface (Jiang et al., 2020; Wang et al., 2017). Under humid conditions, infected leaves often exhibit yellow exudates or amber droplets of bacterial ooze, which can be observed as small beads and serve as a characteristic sign of BLS (Ou, 1985). Conventional management strategies have typically included the application of chemicals and the cultivation of resistant rice varieties. However, the misapplication of pesticides raises significant environmental concerns, and the economic viability of these chemical treatments is often questionable. Furthermore, the emergence of new pathogenic strains, particularly in the context of climate change, suggests that the effectiveness of resistant cultivars may be limited in duration (Fahad et al., 2014). Consequently, the top priority for rice breeding programs needs to focus on developing disease-resistant varieties that not only maintain high productivity but also exhibit durable resistance across strains and environmental conditions (Yadav et al., 2021).

Rice exhibits resistance to BLS as a quantitatively inherited trait; however, the intricacies of the molecular mechanisms governing this resistance remain largely elusive (Tang et al., 2000). To date, a minimum of 20 quantitative trait loci (QTLs) have been identified, which encompass two dominant resistance loci, designated as *Xo1* and *Xo2*, along with three recessive resistance genes: *qBlsr5a*, *bls1*, and *bls2* (Chen et al., 2022; He et al., 2012; Shi et al., 2019; Triplett et al., 2016; Xie et al., 2014). Among these, *qBlsr5a* has undergone fine-mapping through the utilization of sub-chromosome segment substitution lines, thereby revealing *xa5* as a pivotal functional gene that not only confers resistance to BLS but also plays a significant role in mediating resistance to BLB in rice (Xie et al., 2014). By delineating the region of *bls1*, mitogen-activated protein kinase (*OsMAPK6*) was confirmed as a major resistant gene in this QTL region (Ma et al., 2021). Moreover, many studies have also revealed that defense-related genes such as *OsHsp18.0-CI*, *OsWRKY45-2*, *GH3-2*, *OsPGIP1*, *OsHsfB4d*, *OsPSKR1*, *DEPG1*, *OsWRKY45-1*, *OsMPK6*, *NRRB*, *OsBGLU19* and *OsBGLU23*, etc. are involved in defense against BLS infection (Fu et al., 2011; Guo et al., 2014, 2012; Ju et al., 2017; Li et al., 2019; Shen et al., 2010; Tao et al., 2009; Wu et al., 2019; Yang et al., 2020, 2019).

Next-generation sequencing technology-based transcriptome profiling provides comprehensive data regarding candidate genes with expression patterns linked to traits of interest (Gedil et al., 2016). RNA sequencing (RNA-seq) has emerged as a powerful methodology for investigating plant transcriptomes under various stress conditions and across different temporal contexts, yielding high-throughput results (t Hoen et al., 2013). In rice, differential gene expression patterns in response to various abiotic stresses, including arsenic, cadmium, iron, salt, and drought stress (Bashir et al., 2014; Chandran et al., 2019; Sun et al., 2019; Yoo et al., 2017; Yu et al., 2012; Zhang et al., 2017) or pathogens such as blast, black-streaked dwarf virus, sheath blight, root-knot nematode, bacterial leaf blight (Peng Yuan et al., 2020; Wang et al., 2019; Yang et al., 2021; Zhang et al., 2020; Zhou et al., 2020) have been studied by RNA-seq and transcriptome profiling. Earlier studies using RNA-seq have shown the mechanisms underlying BLS disease resistance in rice. A transcriptome study showed that WRKY transcription factors, MAPK signalling pathway, and hormone-related genes are up-regulated in the early stage of BLS infection (Lu et al., 2020), and examination of the molecular mechanism of *xa5* using transcriptional analysis of RNA interference (RNAi) lines revealed that cell death-related genes are key regulators of BLS resistance (Xie et al., 2020).

Our previous studies successfully identified BLS-resistant genes in Thai rice germplasm and highlighted the resistance gene *xa5* as a key factor involved in the response to various *Xoc* isolates (Sattayachiti et al., 2020; Thianthavon et al., 2021). *Xa5*, which was first identified as a BLB-resistance gene encodes the transcription initiation factor IIA gamma subunit 5 (TFIIAγ5) and does not resemble a typical resistance gene immune receptor. *xa5*-based resistance is recessive, and two nucleotide substitutions having a single amino acid change confer either resistance or susceptibility to disease. In plants carrying the susceptible *Xa5* allele, the functional TFIIAγ5 can be exploited by *Xoc’*s Transcription Activator-Like Effectors (TALEs) to activate host susceptibility genes, facilitating disease progression. However, in the resistant *xa5* allele, a specific amino acid change in TFIIAγ5 alters its interaction with *Xoc*’s TALEs, thereby interfering with their ability to efficiently activate these host susceptibility genes (Iyer and McCouch, 2004). This unique mode of action contributes to its durable resistance against various *Xoc* strains. Crucially, in response to *Xoc* infection, the *xa5* allele has been linked to an enhanced induction of host cell death-related pathways, thereby limiting bacterial proliferation and disease progression (Xie et al., 2020). While other transcriptome studies have also mentioned BLS resistance, this study addresses a significant scientific knowledge gap. In our previous GWAS study, it was evident that some rice varieties exhibiting strong resistance to most Thai *Xoc* isolates lack the *xa5* allele or possess other known resistance genes (Sattayachiti et al., 2020). This indicates that an alternative resistance mechanism to *xa5* that can effectively combat BLS must exist, and that these rice varieties could hold the key to the identification of novel resistance strategies and resistance genes. Therefore, this current study was designed (1) to investigate the differences in transcriptomic profiles of rice genotypes with and without *xa5* resistance allele and (2) to identify the alternative mechanism or genes responsible for resistance in the NDCMP49 variety. Here, we employed RNA-seq analysis to identify differentially expressed genes (DEGs) associated with response to *Xoc* infection across three different rice genotypes: DV85, a variety known to harbor the *xa5* and *Xa7* resistance genes for BLB and BLS; Niaw Dam Chaw Mai Pai 49 (NDCMP49), a BLS-resistant variety lacking any known resistance genes; and the BLS-susceptible variety, Hom Cholasit (HCS). For this investigation, transcriptomic data were generated from leaf samples collected following inoculation with *Xoc*, and raw sequencing reads were aligned to the gapless indica reference genome MH63KL1. This RNA-seq analysis identified a range of genes that are potentially important for the activation of pattern-triggered immunity (PTI) and effector-triggered immunity (ETI) pathways, thereby enhancing our understanding of the molecular responses governing disease resistance.

## 2 Materials and methods

### 2.1 Plant materials

DV85 is an *aus* variety from Bangladesh known to encode *xa5* and *Xa7* genes that confer resistance to BLB and BLS (Chen et al., 2021; Sattayachiti et al., 2020). Niaw Dam Chaw Mai Pai (NDCMP49) is a Thai upland rice variety with a distinct multi-spikelet feature from the rachis node, which is resistant to BLS (Nan et al., 2021). Hom Cholasit (HCS) is a Thai improved cultivar that is highly susceptible to BLS, with dominant *Xa5* allele that confers susceptibility to BLS (Sattayachiti et al., 2020). Plant materials were obtained from the germplasm collection maintained by the Rice Science Center, Kasetsart University, Nakhon Pathom, Thailand. Plant growing and inoculation experiments were conducted in the center’s greenhouse facilities from May 2023 to July 2023.

### 2.2 Bacterial strain and growth conditions

For artificial inoculation, gram negative, rod-shaped *Xoc* isolate 1NY2-2, which is one of the representatives of diversity groups from Thailand (Khwanngam, 2015), was kindly provided by Assistant Professor Sujin Patarapuwadol of the Department of Plant Pathology, Faculty of Agriculture at Kamphaeng Saen (Kasetsart University) (**Supplementary Figure S1**). Bacterial inoculum was prepared on peptone sucrose agar (PSA) media at 30°C as described previously by Sattayachiti et al. (2020). A bacterial suspension was prepared in distilled water to a final concentration of 10^8^ cfu/mL (OD_600_ = 0.25).

### 2.3 BLS inoculation

At the active tillering stage, the three youngest fully developed rice leaves of HCS, DV85 and NDCMP49 were inoculated with *Xoc* suspension, using the infiltration method as previously described (Reimers and Leach, 1991; Wonni et al., 2015; Yang and Bogdanove, 2013). A 3-ml needleless syringe was used to infiltrate ~0.5 ml of bacterial suspension from the underside of the leaf by gently pushing the plunger, taking care not to crush the leaves and not to overlap the midrib. Each leaf was infiltrated three times with equal distances between each inoculation point. Two leaves per plant and three plants per genotype were used for inoculation. For the control, the number of inoculated spots and number of plants were the same as bacterial infiltrated plants except distilled water was used for infiltration. The plants were maintained in the greenhouse equipped with a misting system to control humidity (75% relative humidity). Bacterial lesion length was measured at 7 days and 14 days post inoculation (dpi) and average lesion length and standard deviation were calculated. Significant difference was analyzed by one-way analysis of variance (ANOVA) at 0.05 and Fisher’s Least Significant Difference test was performed for pair wise comparison of genotypes by RStudio v 4.3.1.

### 2.4 RNA extraction, quality control, and RNA sequencing

The inoculation was performed between 08.00 - 08.30. Leaf samples were collected between the time of 08.00 – 08.30 for 0 hours post inoculation (hpi) and 17.00 - 17.30 for 9 hpi, as previous findings revealed that a 6-12 hpi interval is a critical period of transcriptional reprogramming for resistance (Cernadas et al., 2014; Gan et al., 2011; Xie et al., 2020). To extract RNA, leaves of all genotypes (HCS, DV85, and NDCMP49) were collected at 2-6 cm away from the inoculation point with *Xoc*, in which the samples were respectively named H0h and H9h for HCS inoculations, D0h and D9h for DV85 inoculations, and N0h and N9h for NDCMP49 inoculations. For control treatment, the leaves were infiltrated with sterilized water, and the samples were named HC0, DC0, and NC0. Three biological replicates were used for every treatment of each genotype. All the collected leaf samples were quickly put into liquid nitrogen and stored at -80°C until use. Total RNA was extracted using TRIzol™ Reagent kit (Invitrogen, USA) according to the manufacturer’s protocol. The total RNA concentration and the RNA integrity in different samples were determined using a NanoDrop spectrophotometer (Thermo Fisher Scientific, USA) and nondenaturing agarose electrophoresis, respectively. Then, the RNA samples were sent to BGI (Shenzhen, China) for library preparation and RNA sequencing according to BGI standard protocols.

### 2.5 RNA-seq data analysis

Generated 150-bp paired-end reads were processed using nf-core/rnaseq v3.12.0 of the nf-core collection of workflows (Ewels et al., 2020). The pipeline was executed with Nextflow v23.04.4 (Di Tommaso et al., 2017) using the default options. Reads alignment and mapping to the gapless indica reference genome MH63KL1 (Li et al., 2021) was performed by Spliced Transcripts Alignment to a Reference (STAR) software v2.7.9a (Dobin et al., 2013) and transcript quantification was executed with SALMON v1.10.1 (Srivastava et al., 2020). The output files from this pipeline were summarized with MultiQC tools v1.14 (Ewels et al., 2016). All the software runs for nf-core/rnaseq and their versions are described in **Supplementary Table S1A**. The differential expression analysis of all the samples was performed in RStudio by using the “edgeR” package v3.42.4. Benjamini and Hochberg’s approach was used to adjust p-values for controlling the false discovery rate (FDR). Genes with the log_2_ fold change (FC) ≥ 1 (up-regulated genes) or ≤ -1 (down-regulated) and adjusted p-value of < 0.05 were designated as differentially expressed.

### 2.6 Functional annotation, gene ontology, and enrichment analysis

Firstly, the transcripts and proteins of the reference genome were annotated by BLAST and DIAMOND search engines against the nt and nr NCBI databases, respectively (Buchfink et al., 2021). In addition to the homology searches, the predicted proteins of the reference genome were annotated using InterProscan (v5.65- 97.0) to assign functionality using Gene Ontology, protein domains, and protein family databases (Jones et al., 2014). GO analysis was performed using clusterProfiler (v4.8.3) in RStudio with an FDR value of 0.05, and GO annotations were grouped into the biological process (BP), cellular component (CC), and molecular function (MF) (Yu et al., 2012). KEGG (Kyoto Encyclopedia of Genes and Genomes) enrichment analysis was carried out by using ShinyGO (v0.77) with an FDR cutoff value of 0.05 (Ge et al., 2020).

### 2.7 Validation of gene expression by qRT-PCR

RNA-seq results were validated by qRT-PCR. The 7 DEGs with their potential role in *Xoc* resistance were randomly selected from H9h vs H0h, D9h vs D0h, N9h vs N0h, D9h vs H9h, N9h vs H9h, and N9h vs D9h. The primers for selected genes were designed by Primer3Plus software, and the primer sequences are listed in **Supplementary Table S1B**. To check the specificity of the designed primers, the primer sequences were blasted again in the NCBI database. As an internal control and for normalization, the constitutively expressed rice actin gene was used. Total RNA extraction was performed by using the TRIzol™ Reagent kit (Invitrogen, USA) according to the manufacturer’s protocol. The first-strand cDNA was synthesized by using iScript™ Reverse Transcription Supermix for RT-qPCR (BioRad, Hercules, CA, USA). Then, quantitative PCR analysis was performed by using KAPA SYBR^®^ Fast qPCR Master Mix (2X) Kit (Kapa Biosystems, USA) and 96-well plates on the Bio-Rad CFX96 real-time system (Bio-Rad, CA, USA). Each reaction has 10 µL of KAPA SYBR FAST qPCR master mix, 1 µL of cDNA sample, and 10 µM of gene-specific primers with a final volume of 20 µL. qRT-PCR reactions were set up as follows: initial denaturation at 95°C for 3 min, followed by 37 cycles of denaturation at 95°C for 10 s, and primer annealing/extension at 60°C for 20 s. Data were evaluated with Bio-Rad CFX ManagerTM (v3.0). The expression levels of selected genes were calculated with 2^-ΔΔCt^. To guarantee the primer-template specificity, melting curve analysis was carried out. For each of the three biological replicates, three technical replicates were made.

### 2.8 Allele mining of candidate genes using whole-genome resequencing data from rice germplasm

The genes selected from RNA-seq analysis according to functional validation and fold change values are further examined for allelic variation in a collection of Thailand’s rice germplasm, which consists of 249 accessions. The *Xoc* inoculation with 1NY2-2 and disease assessment were performed as previously described (Thianthavon et al., 2021). The SNP set used in this study was derived from a whole-genome resequencing project conducted by the National Center for Genetic Engineering and Biotechnology, Thailand, using Nipponbare IRGSP1.0 as the reference genome (Theerawitaya et al., 2022). The SNPs and InDels across the genome present in the selected candidate genes were determined using the GATK software suite (4.6.0.0) and compared among the accessions. The effects of SNPs and InDels were estimated using SnpEff (Cingolani et al., 2012). A genotype-phenotype association analysis was performed on the 249 accessions with a simple regression method using lm() function in R. The genotype data used for the analysis were the SNPs and InDels in the selected candidate genes, and the phenotype data were the BLS scores given 14 days after the *Xoc* inoculation.

## 3 Results

### 3.1 Response to BLS pathogenesis

**Figure 1:**
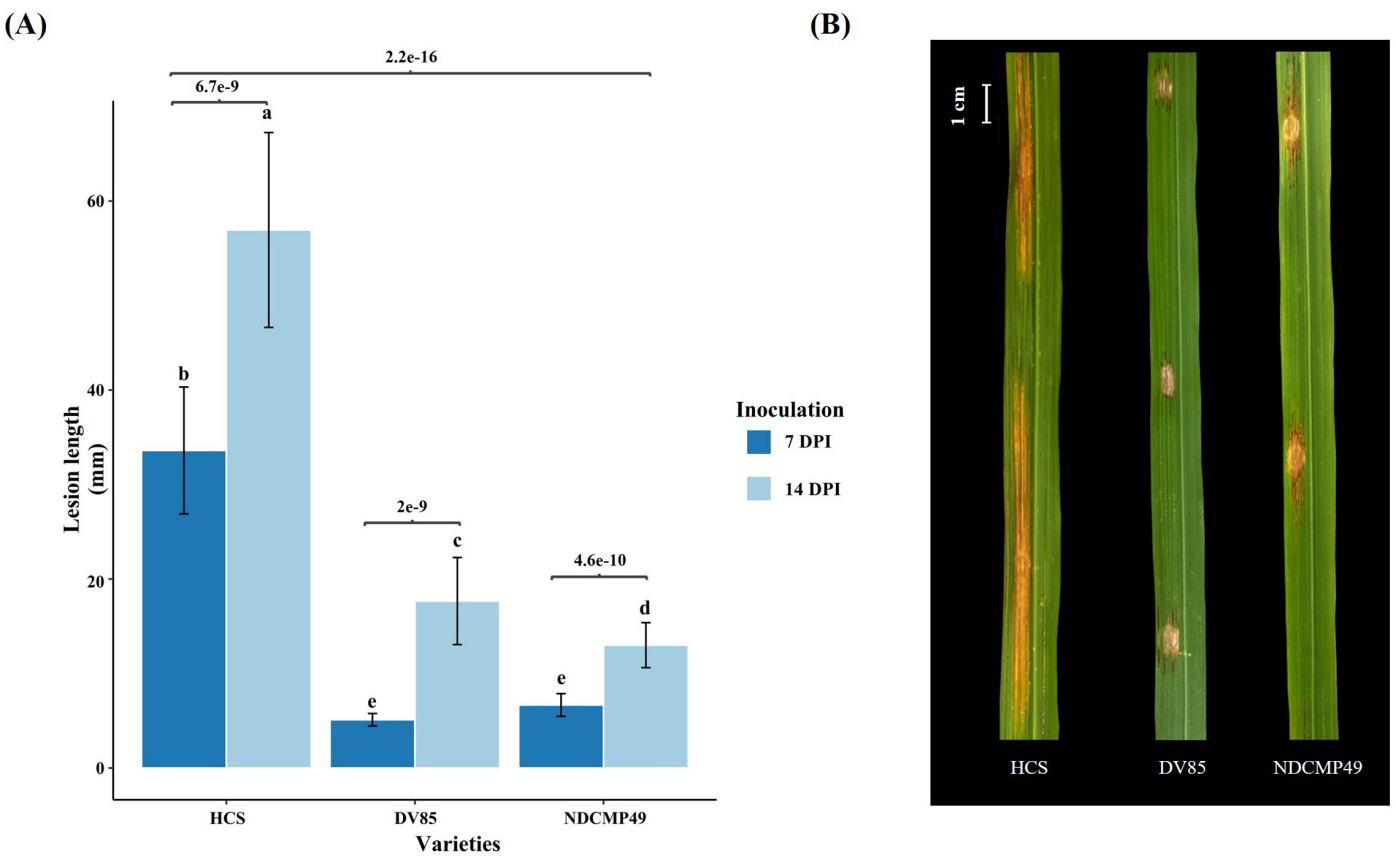
Phenotypic response of HCS, DV85 and NDCMP49 after inoculation with *Xoc*. **(A)** Leaves of all varieties were inoculated with *Xoc* (10^8^ cfu/mL) by injection method at active tillering stage and bacterial lesion length was recorded at 7 days and 14 days after inoculation. Mean and standard deviation were calculated. The statistically significant difference was analyzed by one-way analysis of variance (ANOVA) at 0.05 and pair wise comparison was performed using Fisher’s LSD test by RStudio v 4.3.1. Letters represent the mean comparison values according to Fisher’s LSD test across the genotypes at 7 dpi and 14 dpi. **(B)** Phenotypic images of *Xoc* infected leaves of HCS, DV85 and NDCMP49. Photographs were taken at 14 days of post-inoculation.

To assess the disease response of the two resistant varieties, DV85 and NDCMP49, and a susceptible variety, HCS, we performed an inoculation of a Thai *Xoc* isolate, 1NY2-2, on the leaves at the active tillering stage and recorded the lesion length at 7 and 14 dpi to observe resistance and susceptibility symptoms. Initially, the lesions were small, water-soaked, and mostly confined to the inoculated area. The spreading of lesions was observed at 3 dpi as yellowish linear lesions. For all varieties, lesion lengths were significantly different from 7 dpi to 14 dpi (**Figure 1A**). In resistant varieties, lesions stopped spreading at 14 dpi, with color changing to brown. However, in the susceptible variety, the lesions spread rapidly from both ends of the initial inoculation sites, nearly merging between the injection points at 14 dpi. Yellow beads or bacterial ooze can also be seen on the HCS variety at 14 dpi (**Figure 1B**). According to ANOVA, lesion lengths of all tested genotypes at 7 dpi and 14 dpi showed statistically significant with p-value of 2.2e^-16^. Additionally, the Fisher’s LSD test showed highly significant differences within genotypes for HCS (p = 6.7e^-9^), DV85 (p = 2e^-9^), and NDCMP49 (p = 4.6e^-10^;). According to mean comparison, the lesion lengths of HCS at both 7 dpi and 14 dpi are significantly different from resistant genotypes.

### 3.2 RNA-sequencing statistics and sample distribution

**Figure 2:**
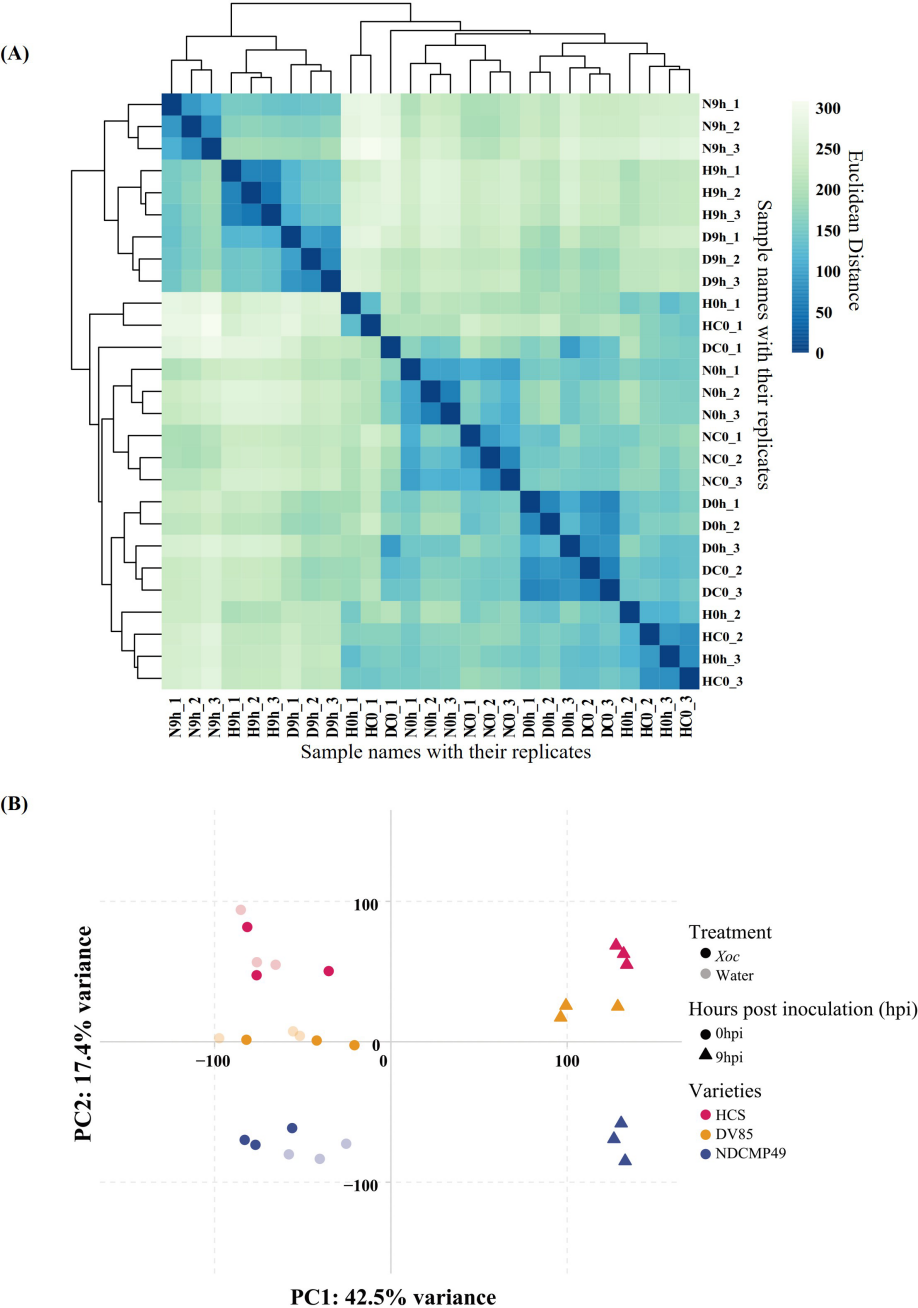
The sample distance matrix and principal component analysis (PCA) of variance stabilized transformed raw counts of all tested samples. **(A)** Sample distance matrix of all biological replicates. **(B)** PC1 shows a 42.5% variance which separates two time points (0hpi and 9hpi) which is shown by different shapes. PC2 exhibits a 17.4% variance of the all-tested genotypes which is shown by different colors. Treated and untreated (control) samples are differentiated by filled and unfilled circles respectively.

Transcriptome profiling was carried out on RNA extracted from *Xoc*-inoculated leaves of HCS, DV85, and NDCMP49 at 0 hpi and 9 hpi. RNA-seq data were exposed as 150 bp pair-end sequencing, resulting in an average of 11.4 GB per sample. The clean reads ranged from 8,267,849 to 19,686,293, and more than 90% of these were uniquely mapped to the MH63KL1 reference genome (**Supplementary Table S1C**). To visualize the sample distribution and similarity of all samples, a sample distance matrix and principal component analysis (PCA) were performed after variance stabilizing transformation (VST) was applied on read counts of all tested samples (**Figure 2**). PCA clustered the three biological replicates of each condition closely to each other. PC1 separated the samples according to hpi, and the variance was 42.5%. PC2 showed the clustering of samples according to genotypes, and the explained variance was 17.4%. *Xoc*-inoculated and mock-inoculated samples at 0 hpi did not significantly separate from each other, indicating that transcriptional changes had not commenced immediately after infiltration with *Xoc* (**Figure 2B**).

### 3.3 Differential gene expression profiles across rice genotypes and time points

**Figure 3:**
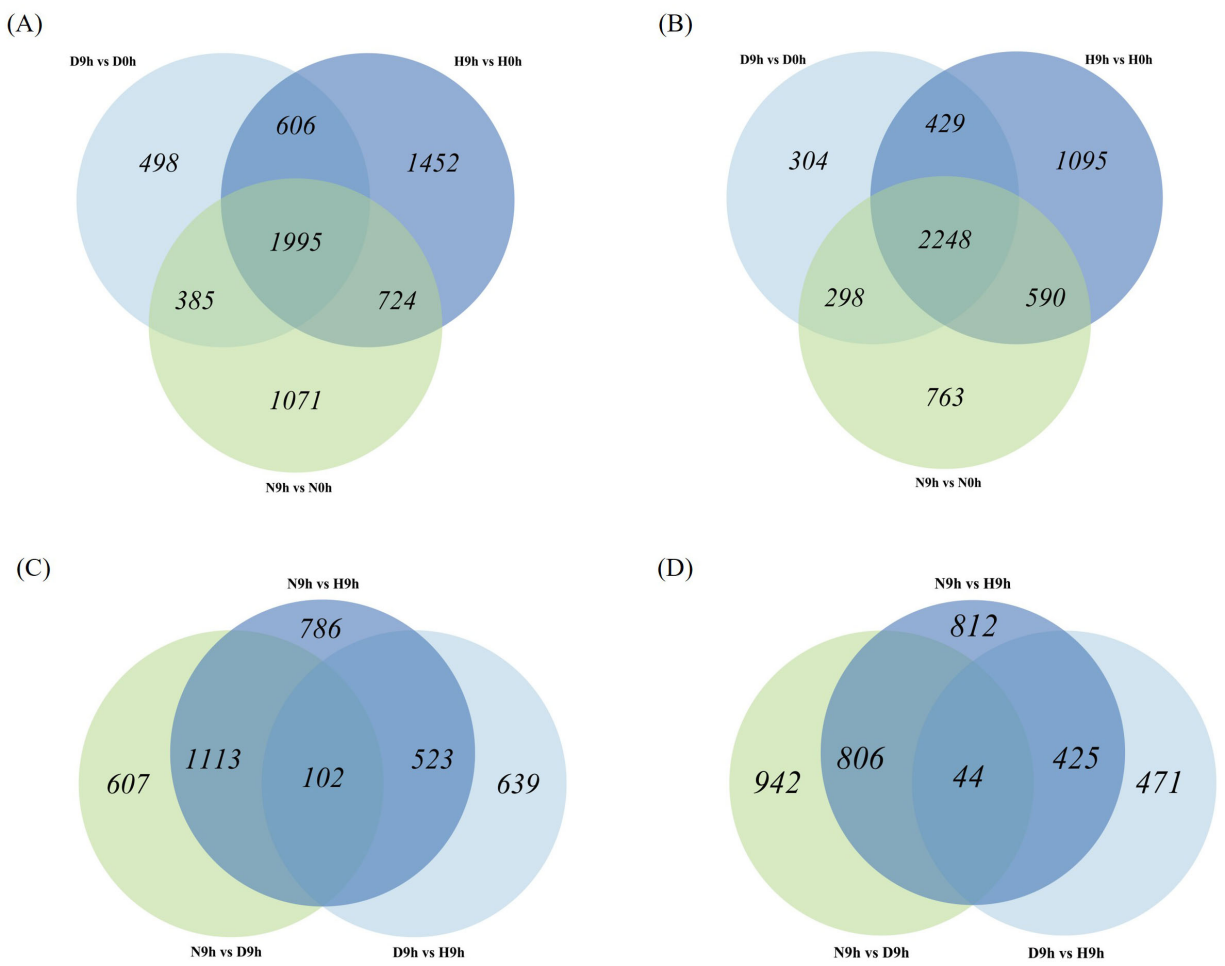
Venn diagrams showing many differentially expressed genes (DEGs) from the RNA-seq of three rice varieties (HCS, DV85, and NDCMP49). **(A)** Venn diagram showing up-regulated DEGs overlapping between all genotypes at 9 hpi compared to 0 hpi. **(B)** Venn diagram illustrating down-regulated DEGs overlapping between all genotypes at 9 hpi compared to 0 hpi. **(C)** Venn diagram showing up-regulated DEGs when genotypes are compared to each other at 9 hpi by subtracting 0 hpi. **(D)** Venn diagram depicting down-regulated DEGs when genotypes are compared to each other at 9 hpi by subtracting 0 hpi.

EdgeR package analysis was applied to identify differentially expressed genes (DEG) among different rice genotypes and time points. Overall, a total of 35,760 DEGs were identified across the samples. After the application of log_2_fold change (log_2_FC) ≥1 or ≤-1 and p-value (FDR) of 0.05 thresholds, 19,438 DEGs remained, which were analyzed further. Paired comparisons were carried out on H9h vs H0h, D9h vs D0h, N9h vs N0h, D9h vs H9h, N9h vs H9h, and N9h vs D9h to investigate expression patterns of DEGs at different time points and in differing genotypes (**Supplementary Table S2**). In HCS, a total of 4,777 DEGs were upregulated and 4,362 DEGs were downregulated in H9h compared to H0h. In DV85, 3,484 DEGs were upregulated and 3,279 DEGs were downregulated in D9h compared to D0h, and in NDCMP49, 4,175 DEGs were upregulated and 3,899 DEGs were downregulated in N9h compared to N0h (**Figures 3A, B**). When the genotypes were compared to each other at 9 hpi, 1,264 and 940 DEGs were upregulated and downregulated, respectively, in D9h vs H9h. In N9h vs H9h, 2,524 DEGs were upregulated while 2,087 DEGs were downregulated. In resistant genotypes, N9h vs D9h, 1,822 and 1,792 DEGs were upregulated and downregulated, respectively (**Figures 3C, D**). There were significantly more DEGs in the genotype-genotype pair of N9h vs H9h, suggesting a more widespread transcriptional response in the disease resistance genotype, NDCMP49, and the susceptible genotype, HCS, at the early infection stage.

### 3.4 GO enrichment analysis of the identified DEGs

**Figure 4:**
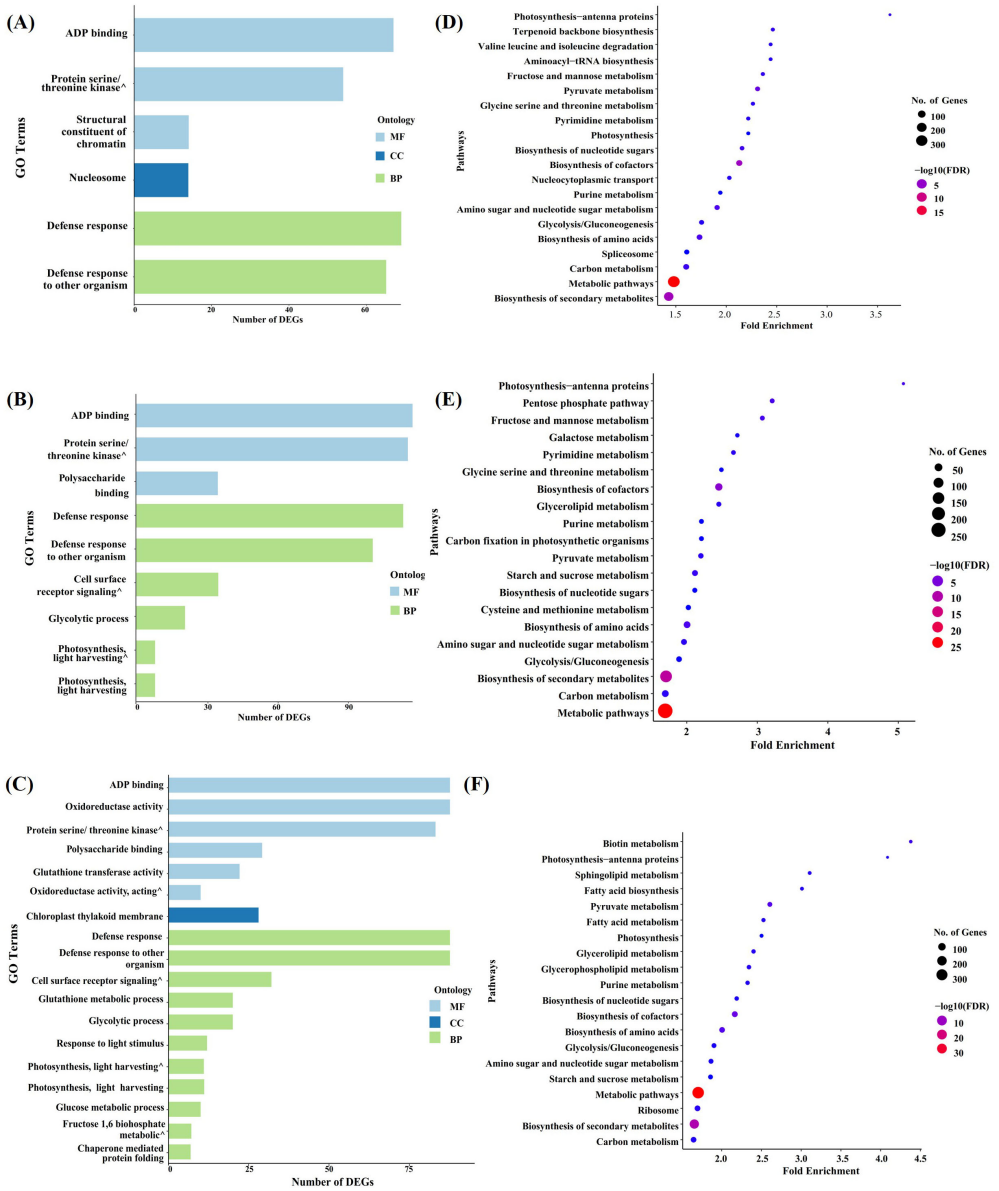
GO enrichment and KEGG pathway enrichment analyses of DEGs. **(A)** The Bar graph shows GO categories that are significantly enriched in D9h vs H9h. **(B)** The bar graph illustrates GO categories that are enriched in N9h vs H9h. **(C)** The bar graph gives GO terms enriched in N9h vs D9h. (The x-axis illustrates the number of DEGs in each GO term and the y-axis describes the enriched GO categories. MF= Molecular Function, CC= Cellular Component, BP= Biological Process). **(D)** Bubble diagram expresses KEGG enrichment analysis of H9h vs H0h. **(E)** Bubble diagram shows the KEGG enrichment analysis of D9h vs D0h. **(F)** Bubble diagram illustrates KEGG enrichment analysis of N9h vs N0h. (X-axis represents the fold enrichment factor and y-axis describes the pathways. The bubble size shows the number of DEGs that are enriched in a particular pathway and the color of the bubbles expresses the negative log10 values (the significance of the enrichment).

Gene Ontology (GO) enrichment analysis was used to characterize the molecular components and pathways involved in the response of the three genotypes to *Xoc*. Paired comparisons of D9h vs H9h, N9h vs H9h, N9h vs D9h, H9h vs H0h, D9h vs D0h, and N9h vs N0h were carried out using a FDR ≤ 0.05. This assigned the DEGs into three main functional groups, namely, biological process (BP), cellular component (CC), and molecular function (MF), as shown in **Supplementary Table S3**. In the paired comparison, D9h vs H9h, 283 DEGs were grouped into 6 GO functional terms (**Figure 4A**). Most of these DEGs enriched are under the GO aspect BP; 134 DEGs were involved in defense response and defense response to other organisms. Under the CC aspect, 14 DEGs were over-represented in the category of nucleosome. Under the MF aspect, 135 DEGs were categorized as ADP binding, structural constituent of chromatin, and protein serine/threonine kinase activity. In the paired comparison between N9h vs H9h, a total of 556 DEGs were over-represented across 9 functional groups (**Figure 4B**). The majority of these DEGs were associated with the GO aspect BP, and their categories included defense response, defense response to other organisms, cell surface receptor signaling pathway, and photosynthesis (light harvesting in photosystem I), etc. Under the MF aspect, 147 DEGs were over-represented in N9h, and the enriched categories were ADP binding, protein serine/threonine kinase activity, and polysaccharide binding. It was clear that, overall, both resistant genotypes showed enrichment of more DEGs related to defense mechanisms against the pathogen than the susceptible genotype. In the paired comparison between the two BLS-resistant genotypes at 9 hpi, N9h vs D9h, a total of 680 DEGs were associated with 18 GO terms (**Figure 4C**). In the BP aspect, 322 DEGs were enriched in 11 enrichment terms, such as defense response to other organisms or defense response. Only 28 DEGs were enriched under the CC aspect, and these were all associated with the GO term chloroplast thylakoid membrane. In the MF aspect, 330 DEGs were grouped into 6 GO terms, the most enriched terms being ADP binding and oxidoreductase activity.

The comparisons between the two timepoints might be expected to show DEGs related to circadian-regulated genes in addition to those related to *Xoc* infection. In comparing H9h vs H0h, 1,877 DEGs were categorized into 40 different functional groups, 470 DEGs were associated with the BP aspect, 624 with the CC, and 783 with the MF aspect. Most of the DEGs were enriched within 14 MF terms, notably zinc ion binding and catalytic activity (**Supplementary Figure S2**). In D9h vs D0h, 1011 DEGs were enriched in 22 functional GO terms, 10 within BP, 4 within CC, and 8 within MF aspects. The 424 DEGs related to MF were most enriched in zinc ion binding and catalytic activity terms (**Supplementary Figure S2**). In the paired comparison between N9h vs N0h, 1,543 genes were enriched for 26 GO terms; 378 DEGs were associated with 10 GO terms within BP, 685 DEGs were associated with 9 GO terms within CC, and 480 DEGs were associated with 7 GO terms within MF. Thus, DEGs in the CC aspect appear to be a major contributor in NDCMP49’s response to *Xoc*. The majority of these DEGs were associated with the GO terms chloroplast, mitochondrion, and ribosome (**Supplementary Figure S2**).

Several enriched GO terms were shared between genotypes at 9 hpi. Under BP, GO terms including zinc ion binding, catalytic activity, GTP binding, pyridoxal phosphate binding, and ATP-dependent peptidase activity were identified across all three genotypes. Within the CC aspect, chloroplast, intracellular membrane-bounded organelle, chloroplast thylakoid membrane, and chloroplast stroma GO terms were commonly annotated between genotypes. Within the MF aspect, signal transduction, RNA modification, protein folding, photosynthesis/light harvesting, and photosynthesis/light harvesting in photosystem I terms were commonly enriched between genotypes. Perhaps more important than the similarities between genotypes (that may be attributable to factors in common, such as circadian effects or wound responses) are the differing responses between the genotypes. GO terms that are differently enriched between the three genotypes might be expected to contribute to their differing susceptibility or resistance defense mechanisms. In this respect, it was notable that NDCMP49 had more DEGs at 9 hpi, which were enriched for GO terms such as defense response and defense response to other organisms in comparison to the other two genotypes. The identification of these DEGs represents the first step in characterizing the previously unknown response system that this variety employs to resist infection by the *Xoc* pathogen.

### 3.5 Pathway analysis of differentially expressed genes by KEGG analysis

Analysis of pathways involved in genotype responses to *Xoc* infection was carried out using the Kyoto Encyclopedia of Genes and Genomes (KEGG) database, based on a p-value of ≤ 0.05. The analysis was done on pair comparisons: H9h vs H0h, D9h vs D0h, N9h vs N0h, N9h vs H9h and N9h vs D9h. A comparison between H9h and H0h DEGs, 907 DEGs identified 20 pathways as being involved in **Supplementary Table S4**. Among these enriched pathways, metabolic pathways, biosynthesis of secondary metabolites, biosynthesis of cofactors, pyruvate metabolism, amino sugar and nucleotide sugar metabolism, biosynthesis of metabolism, and carbon metabolism are highly enriched (**Figure 4D**). Comparison between D9h and D0h identified 751 DEGs in 20 pathways (**Figure 4E**, **Supplementary Table S4**), including metabolic pathways, biosynthesis of secondary metabolites, biosynthesis of cofactors, and biosynthesis of amino acids with the highest -log10 value. The majority of genes were enriched in metabolic pathways and biosynthesis of secondary metabolites, with DEGs of 269 and 256, respectively. In the N9h vs N0h comparison, 931 DEGs were associated with 20 pathways, including metabolic pathways, carbon metabolism, biosynthesis of cofactors, biosynthesis of amino acids, pyruvate metabolism, and biotin metabolism with the highest -log10 values (**Figure 4F**, **Supplementary Table S4**). The most enriched terms, metabolic pathways, and biosynthesis of secondary metabolites had 336 and 188 DEGs associated, respectively. Again, several pathways, including biosynthesis of secondary metabolites, metabolic pathways, and biosynthesis of cofactors, were commonly enriched across all three genotypes at 9 hpi. These may be involved in the response of all genotypes against *Xoc* infection, or perhaps in unrelated processes such as circadian-regulated events. When NDCMP49 was compared to HCS and DV85 at 9 hpi, both comparisons showed 9 KEGG pathways that passed a p-value of 0.05, and the majority of DEGs were enriched in metabolic pathways, with 158 DEGs and 133 DEGs, respectively (**Supplementary Figure S3**). Surprisingly, the KEGG analysis identified no enriched terms in the comparison between the resistant and susceptible varieties D9h vs H9h.

### 3.6 Modulation of genes associated with pathogen recognition

Nine hours after *Xoc* inoculation, several gene classes related to pathogen detection and recognition, signal transduction, and defense response were differentially expressed. Among them, 132 DEGs related to receptor-like kinases (RLKs) were identified, including leucine-rich receptor-like kinases (LRR-RLKs), receptor-like cytoplasmic kinases (RLCKs), wall-associated kinases (WAKs), malectin-like receptor-like kinases (MRLKs), somatic embryogenesis receptor kinases (SERKs), and S-domain receptor-like kinases (SDRLKs). These receptor-like kinase family members have been reported to have roles in pathogen detection and recognition (Chen et al., 2014; Malukani et al., 2020; Ortiz-Morea et al., 2022; Song et al., 1995; Zhou et al., 2016) Hence, their identification here indicates that they also play a role in plant defense against *Xoc*.

Hierarchical clustering of the RLKs illustrated that different RLKs show up- and down-regulation between 0 hpi and 9 hpi (**Supplementary Figure S4** and **Supplementary Table S5**). In H9h vs H0h, 70 RLKs passed the fold change threshold (log_2_FC ≤-1 or ≥1) with log_2_FC values ranging from -5.6 to 6.1. In this comparison the most down-regulated RLKs included *OsRLCK303*, *OsMAK10*, *OsRLCK269*, *OsRLCK164* and *OsRLCK255*, and the most up-regulated RLKs included *OsRLCK233*, *OsRLCK30*, *OsRLCK350*, *OsWAK123* and *OsRLCK358*. In the D9h vs D0h comparison, the fold change values ranged from -4.5 to 5.8, and RLKs including *OsMAK10*, *OsRLCK164*, *OsLecRK* paralog, *OsWAK107* were down-regulated, whilst *OsRLCK358*, *OsRLCK233*, *OsWAK107*, and *OsRLCK29* were up-regulated. In N9h vs N0h, the log_2_FC values ranged from -6.2 to 6.7, and RLKs including *OsRLCK164*, *OsRLCK343*, and *OsWAK107* showed significant downregulation, and *OsFbox591*, *OsRLCK233*, cystine-rich receptor-like kinase 6, *Gnk2RLK-4*, and *OsFbox591* were highly up-regulated. In the D9h vs H9h comparison, 44 genes were differentially expressed with log_2_FC values from -9.03 to 7.96. Highly downregulated DEGs included *OsMRLK61*, *OsSDS2*, *OsMCA1* and *OsRLCK11*, and highly up-regulated DEGs included *OsMRLP16*, *OsMRLK61*, *OsRLCK22* and *OsWAK83*. Interestingly, another major BLB resistance gene, the *RLK Xa21*, showed significant up-regulation in D9h with a log_2_FC value of 4.65. In the paired comparison of N9h vs H9h, 62 genes were differentially expressed with log_2_FC threshold values ranging from -6.9 to 13.44. The most significant down-regulated DEGs included *OsMRLK61*, *OsMRLK4*, *OsMRLK6*, *OsRLCK11*, and *OsRLCK350*, and up-regulated DEGs included *OsWAK112*, *OsWAK83*, *OsWAK80*, *OsLRR-RLK2* and *Xa21*. Comparing the response of the two resistant genotypes, N9h vs D9h, identified 53 DEGs with log_2_FC values from -8.6 to 9.9. The highly down-regulated DEGs included *OsMRLK61*, *OsWAK107*, *OsRLCK21*, and *OsRLCK22*, and the highly up-regulated DEGs included *OsWAK112*, *OsSDS2*, *OsRLCK35*, *OsLRR-RLK2* and *Xa21*.

### 3.7 Differential expression of transcription factors after *Xoc* infection

Following *Xoc* inoculation, 116 transcription factor genes with roles in the response to pathogens were differentially expressed. The differential expressions of these 31 WRKY, 24 NAC, 34 bHLH, and 27 MYB family transcription factors are shown in **Supplementary Figure S4** and **Supplementary Table S6**. In all varieties, most transcription factors show stronger up-regulation at 0 hpi than at 9 hpi, but DV85 and HCS had the most similar expression patterns, and this included some commonality between differentially expressed transcription factors. In D9h vs D0h, 63 DEGs passed the threshold, with log_2_FC values ranging from -10.47 to 4.72. The most significantly downregulated transcription factors included *OsNAC14*, *OsWRKY108* and *OsWRKY76*, and up-regulated ones included *OsMYB108*, *OsWRKY30*, *OsbHLH045*, *OsbHLH091*, *OsWRKY14*, *OsMYB14* and *OsWRKY88*. In H9h vs H0h, there were 74 DEGs with log_2_FC from -10.47 to 4.22. *OsWRKY76*, *Os2R_MYB6*, *OsWRKY108*, *OsNAC14*, *OsWRKY104*, *OsbHLH185* and *OsWRKY21* showed significant down-regulation while *ONAC063*, *OsMYB108*, *OsbHLH045*, *OsWRKY47* and *OsWRKY88* were up-regulated. In N9h vs N0h, there were 69 DEGs with threshold values ranging from 8.85 to 5.93. *ONAC077*, *OsWRKY14*, *OsMYB108*, *OsWRKY50*, *OsWRKY77*, *OsMYB108*, *OsbHLH037*, and *ONAC062* were highly up-regulated and *OsWRKY104*, *OsbHLH185*, *OsHLH61*, *OsMYBR17*, *ONAC131* and *OsMYB22* were the most down-regulated. Genotype-to-genotype comparisons were also analyzed at 9 hpi. In D9h vs H9h, only 10 transcription factors were differentially expressed with log_2_FC values from -5.3. Down-regulated genes included *OsbHLH012*, *OsDLN4* and *OsWRKY88* and up-regulated *OsWRKY76*, *OsbHLH164*, *OsWRKY62* and *OsWRKY30*. When N9h was compared to H9h, 41 transcription factors were differentially regulated, and the log_2_FC threshold ranged from -5.63 to 8.03. *OsbHLH097*, *OsbHLH012*, and *OsbHLH045* were the most down-regulated and *OsWRKY76*, *OsWRKY62*, *OsWRKY50*, *OsWRKY77* up-regulated in NDCMP49. In the pairwise comparison of N9h vs D9h, 20 DEGs passed the threshold values for log_2_FC and FDR. *OsbHLH097*, *OsWRKY30*, *OsbHLH117*, and *OsWRKY125* were the most down-regulated transcription factors, and *OsWRKY77*, *OsbHLH187*, *OsNAC077*, and *OsWRKY62* were the most up-regulated DEGs in N9h.

### 3.8 Expression of R-gene-mediated and defense-related genes

**Figure 5:**
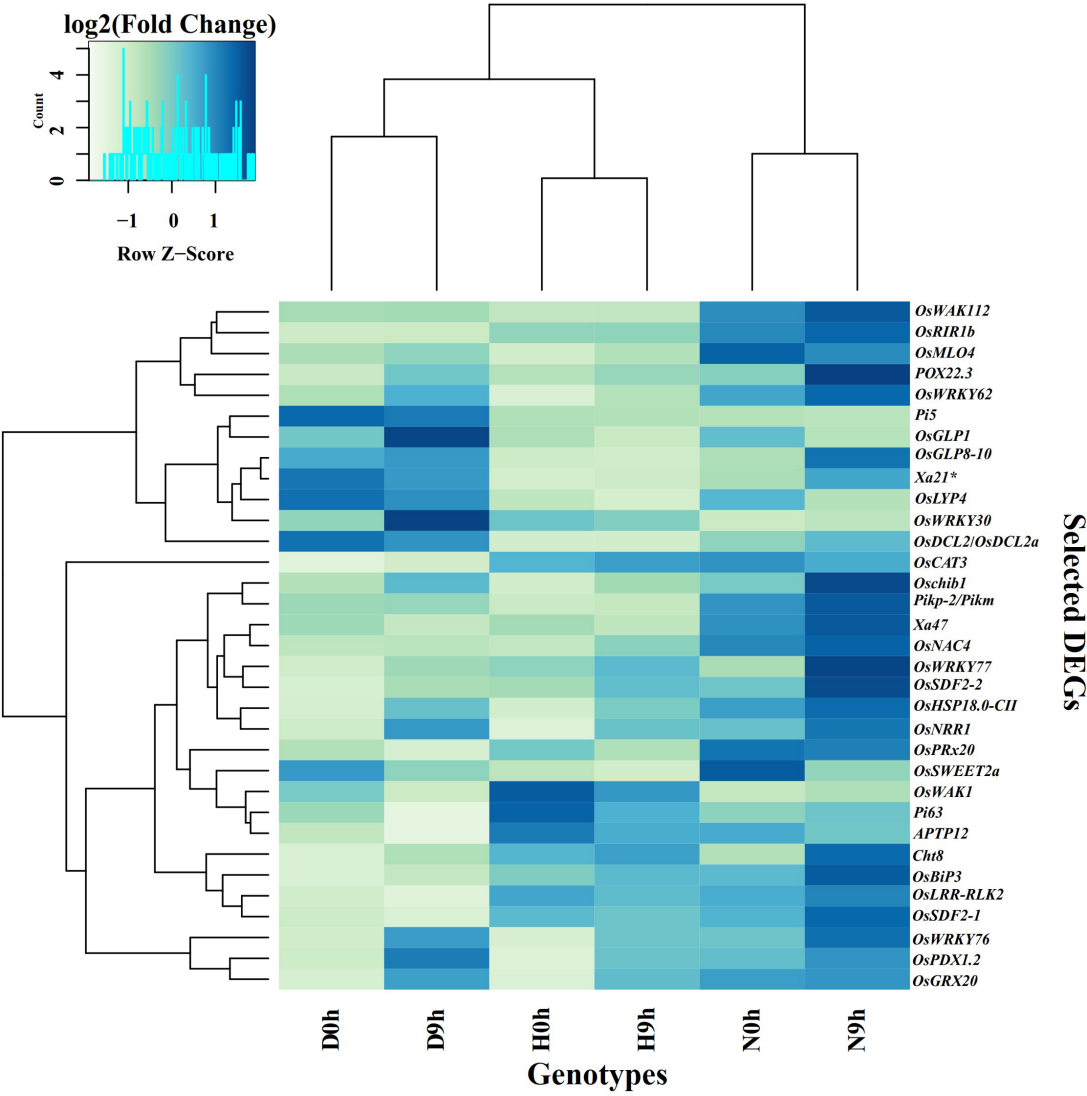
Hierarchical clustering of important DEGs, which are important in pathogen detection, signal transduction, and defense response, of all genotypes at 0 hpi and 9 hpi. Heatmap showing the expression profile of some important DEGs which are selected from categories of receptor-like kinases (RLKs), which play crucial roles as pattern recognition receptors (PRR) in plant immunity, transcription factors (TFs) in which the majority are involved in transcription of downstream genes related with defense response to pathogen and send signals for downstream pathways and other important proteins that have essential roles in signal transduction to initiate defense response and resistance. (Data corresponds to Trimmed Mean of M value (TMM). Z-scores and color key represent the scaled expression values, with blue for low expression and light green to yellow for high expression. Clustering of genes is shown on the left side of the figure and gene names are shown on the right.

In addition to RLKs and transcription factors, other DEGs that are related to disease resistance, such as genes encoding enzymes, heat shock proteins, hormones, and peroxidases, nucleotide binding (NB-LRR) domain, and pentatricopeptide repeats (PPR) proteins were also detected (**Supplementary Figure S4** and **Supplementary Table S7**). In H9h vs H0h, there were 75 such DEGs with fold change values from -10.46 to 6.64. The most significant down-regulated DEGs included *OsSWEET2b*, *OsDEFR3*, *OsUGT74H4*, *OsMAP3K6*, *OsJAZ13* and *OsNPK1-PK*, while the up-regulated genes included *OsPRX126*, *OsPLS19*, *OsHsfC1b* and *OsGRDP1*. In D9h vs D0h, 67 genes were differentially expressed with log_2_FC threshold values from -10.37 to 7.67. The most down-regulated genes included *OsDERF3*, *OsNPK1-PK*, *OsJAZ13*, *OsUGT74H4* and *OsMAPKKK63*, and upregulated *OsHsfC1b*, *OsPrx114*, *OsPrx126* and *OsPrx115*. In N9h vs N0h, 80 genes were differentially expressed with fold change threshold values ranging from -9.52 to 7.77. *OsDERF3*, *OsMAPKKK63*, *OsMAP3K6*, *OsCAO*, *OsJAZ13* and *OsCP26* were the most significantly down-regulated and *OsHsfC1b*, *OsPrx114*, *POX22.3* and *OsChia2a* were the most up-regulated genes. In the genotype-to-genotype comparison D9h vs H9h, 47 DEGs reached log_2_FC values ranging from -9.56 to 11.28. The most significant down-regulated DEGs included *OsNBDGO35*, *RGA5*-like protein, *Pik-2-like* gene, and *Pi63*, while the up-regulated included *OsDCL2a*, *RGA5*-like protein, *Pi3/Pi5-1*, and *OsPDX1.2*. In N9h vs H9h, 67 DEGs had FC values of -9.56 to 10.6. The down-regulated DEGs were *OsNBSGO35*, *RGA5*-like protein, *OsRYMV3* and *PIC27*, and up-regulated were *Osprx114*, *POX22.3*, *OsDCL2a*, *Oschib1*, *Pik-m/Pik-2* and *OsHSP18.0-CII*. When the two resistant genotypes were compared in N9h vs D9h, 69 DEGs with FC expression values of -8.85 to 14.5 were identified. Significantly downregulated DEGs included *RGA3*, *RGA1*, *Pi3/Pi5-1*, *RPP13* and *OsVQ34*, while up-regulated included *OsRALF-30*, *RGA4*, *Pik-2* like protein, *OsHSP18.0-CII* and *Pi63*. Interestingly, some DEGs such as *Pi63*, *Xa22*, *Xa47*, and *OsMLO4*, identified under this category, have previously been reported as major contributing genes responsible for BLB, blast disease, and powdery mildew disease. The most notable DEGs from all the categories with annotations indicating functions in pathogen resistance in rice are listed in **Table 1**, and their normalized transcript counts are described in the hierarchical clustering plot (**Figure 5**).

**Table 1:** Selected DEGs with their functional annotation and fold change expression values.

No.	Gene name/ID	Gene annotation and functions	D9h vs H9h	N9h vs H9h	N9h vs D9h
1	*OsNRR1* (Os01g0130200)	Negative regulator of resistance. Overexpression affects resistance against *Xoo* (Chern et al., 2005).	3.3	6.14	2.84
2	*OsHSP18.0-CII* (Os01g0184100)	18.0 kDa class II heat shock protein. Resistance to bacterial leaf blight disease (Kuang et al., 2017).	N/A	6.25	7.35
3	*APIP12* (Os01g0383900)	AvrPiz-t interacting protein 12. M. *oryzae* effector target gene (Tang et al., 2017).	N/A	N/A	-1.01
4	*OsSWEET2a* (Os01g0541800)	SWEET sugar transporter. Negative regulation of sheath blight resistance (Gao et al., 2021).	N/A	N/A	-2.23
5	*OsWRKY77* (Os01g0584900)	WRKY transcription factor 77. Resistance against bacteria (Lan et al., 2013).	N/A	5.23	7.26
6	*OsNAC4* (Os01g08161000)	NAC domain-containing transcription factor 4. Positive regulator of plant cell death (Kaneda et al., 2009).	-4.9	1.84	6.74
7	*OsPRX20* (Os01g0962700)	Peroxidase. Mediate ROS accumulation (Blackman and Hardham, 2008).	N/A	2.62	1.79
8	*OsBiP3* (Os02g0115900)	ER-localized chaperone. Regulation of *Xa21* protein stability and resistance to *Xoo* (Park et al., 2010).	N/A	4.52	3.22
9	*OsCAT3* (Os03g0131200)	Catalase. Promotion of cell death, ROS scavenging, defense against pathogen (Jiang et al., 2023).	0.93	-0.95	-1.88
10	*OsDCL2a*/*OsDCL2* (Os03g0583900)	Dicer like proteins. Triggered by Southern rice black-streaked dwarf virus (Xu and Zhou, 2017).	11.28	9.91	-1.36
11	*Pi63* (Os04g0620950)	NBS-LRR domain-containing protein. Resistance to rice blast disease (Xu et al., 2014).	-6.5	N/A	6.31
12	*POX22.3* (Os07g0677200)	Peroxidase. Regulation upon rice neck blast disease and *Xoo* infection (Hao et al., 2012).	4.32	10.28	5.96
13	*OsGLP8-10* (Os08g0189900)	Germin-like protein 8-10. Resistance against blast disease (Banerjee and Maiti, 2010).	3.33	5.53	2.2
14	*OsSDF2-1* (Os08g0278900)	Stromal cell-drive factor 2, Endoplasmic reticulum-quality control (ER-QC) protein. *Xa21*-mediated resistance to *Xoo* (Park et al., 2013).	N/A	2.52	2.5
15	*OsSDF2-2* (Os08g0440500)	Stromal cell-drive factor 2, Endoplasmic reticulum-quality control (ER-QC) protein. *Xa21*-mediated resistance to *Xoo* (Park et al., 2013).	N/A	2.92	2.16
16	*OsGLP1* (Os08g0460000)	Germin-like protein 1. Response to sheath blight and blast diseases (Manosalva et al., 2009).	N/A	N/A	-6.7
17	*OsWRKY30* (Os08g0499300)	WRKY transcription factor 30. Positive regulator of rice disease resistance via SA signaling pathway (Han et al., 2013).	4.15	N/A	-5.3
18	*OsGRX20* (Os08g0558200)	Glutaredoxin 20. Positive regulator of rice resistance to BLB (Ning et al., 2018).	3.07	1.63	-1.44
19	*Pi5* (Os09g0327575)	CC-NBS-LRR domain-containing protein. Blast disease resistance (Lee et al., 2009).	5.49	N/A	-5.52
20	*OsWRKY76* (Os09g0417600)	WRKY transcription factor 76. Resistance against rice blast fungus infection (Peng et al., 2010).	4.81	8.03	N/A
21	*OsWRKY62* (Os09g0417800)	WRKY transcription factor 62. Resistance against rice blast and leaf blight disease (Peng et al., 2010).	4.22	7.4	3.18
22	*OsLYP4* (Os09g0452200)	Lysin motif-containing protein. Pattern recognition receptors (Liu et al., 2012).	N/A	N/A	-3.23
23	*OsPDX1.2* (Os10g0100700)	Pyridoxal phosphate synthase 1.2. Overexpression shows resistance against *Xoc* (Liu et al., 2022).	5.48	3.34	-2.14
24	*OsWAK112* (Os10g0180800)	Wall-associated receptor-like protein kinase112. Negative regulator of blast disease resistance (Delteil et al., 2016; Lin et al., 2021).	N/A	13.44	9.91
25	*Oschib1* (Os10g0416500)	Class IIIb chitinase, Family 8 of plant pathogenesis-related protein. Growth inhibition of pathogenic fungi (Tanaka et al., 2023).	5.19	8.16	2.97
26	*OsMLO4* (Os10g0541000)	Powdery Mildew Resistance (Nguyen et al., 2016).	N/A	4.3	3.26
27	*Cht8* (Os10g0542900)	Pathogenesis related-3 chitinase 8 (Fan et al., 2014).	N/A	4.22	2.96
28	*RIR1b* (Os10g0569800)	Rapid alkalization factor 30 (Schaffrath et al., 2000).	-8.15	6.37	14.52
29	*Xa21* (Os11g0559200)	Protein kinase, core domain-containing protein. Resistance to bacterial leaf blight disease (Song et al., 1995).	4.65	6.71	2.06
30	*Xa47* (Os11g0688832)	Coiled-coil NBS-LRR protein. Resistance to rice bacterial leaf blight (Lu et al., 2022).	N/A	2.73	3.16
31	*Pik-2/Pik-m* (Os11g0689100)	CC-NBS-LRR protein. Blast disease resistance (Zhai et al., 2011).	N/A	7.53	6.92
32	*OsWAK1* (Os11g0690066)	Wall-associated receptor-like protein kinase1. Resistance to rice blast disease (Li et al., 2009).	-4.78	N/A	3.88
33	*OsLRR-RLK2* (Os12g0210400)	leucine-rich repeat and receptor-like kinase domain protein 2. Repressor of rice resistance to *Xoo* infection (An et al., 2022).	N/A	2.4	2.71

### 3.9 Validation of differential gene expression by quantitative real-time PCR

Differential expressions of DEGs detected by RNA-seq were validated by using quantitative real-time PCR (qRT-PCR) analysis, and the same RNA extracts were used for RNA-seq experiments. For the verification of the accuracy, 7 DEGs were randomly selected according to their potentially important functions in disease resistance. From each rice genotype, all conditions were used for qRT-PCR analysis, and the internal control was used for the normalization of the Ct values. The relative fold change of selected DEGs was calculated and plotted along with the differential expression values from the RNA-seq analysis (**Supplementary Figure S5**). Our analysis showed a substantial agreement of up- and down-regulation between RNA-seq and qRT-PCR analyses.

### 3.10 Candidate genes with distinct variants in a rice diversity panel exhibiting different phenotypes against BLS disease

We further examined the candidate genes to understand if these genes have variation in the diversity panel. A total of 249 rice accessions were used for phenotyping, and their disease scores can be seen in **Supplementary Table S8**. By using whole-genome re-sequencing data, a total of 1,208 variants, such as SNPs and InDels were detected for the selected candidate genes from RNA-seq analysis, and they were compared among all rice accessions (**Supplementary Table S9**). Among these variants, 214 variants with high confidence were identified not only in the coding region but also in the regulatory sequences. As a result, we found that *OsBIP3*, *POX22.3*, *OsSDF2-1*, *OsSDF2-2*, *RIR1b*, and *OsLRR-RLK2* have several significant variants (p-value <0.05) among the accessions (**Supplementary Table S9**). Subsequently, these variants were used to perform genotype-phenotype association tests with disease scores determined in 249 rice accessions.

**Figure 6:**
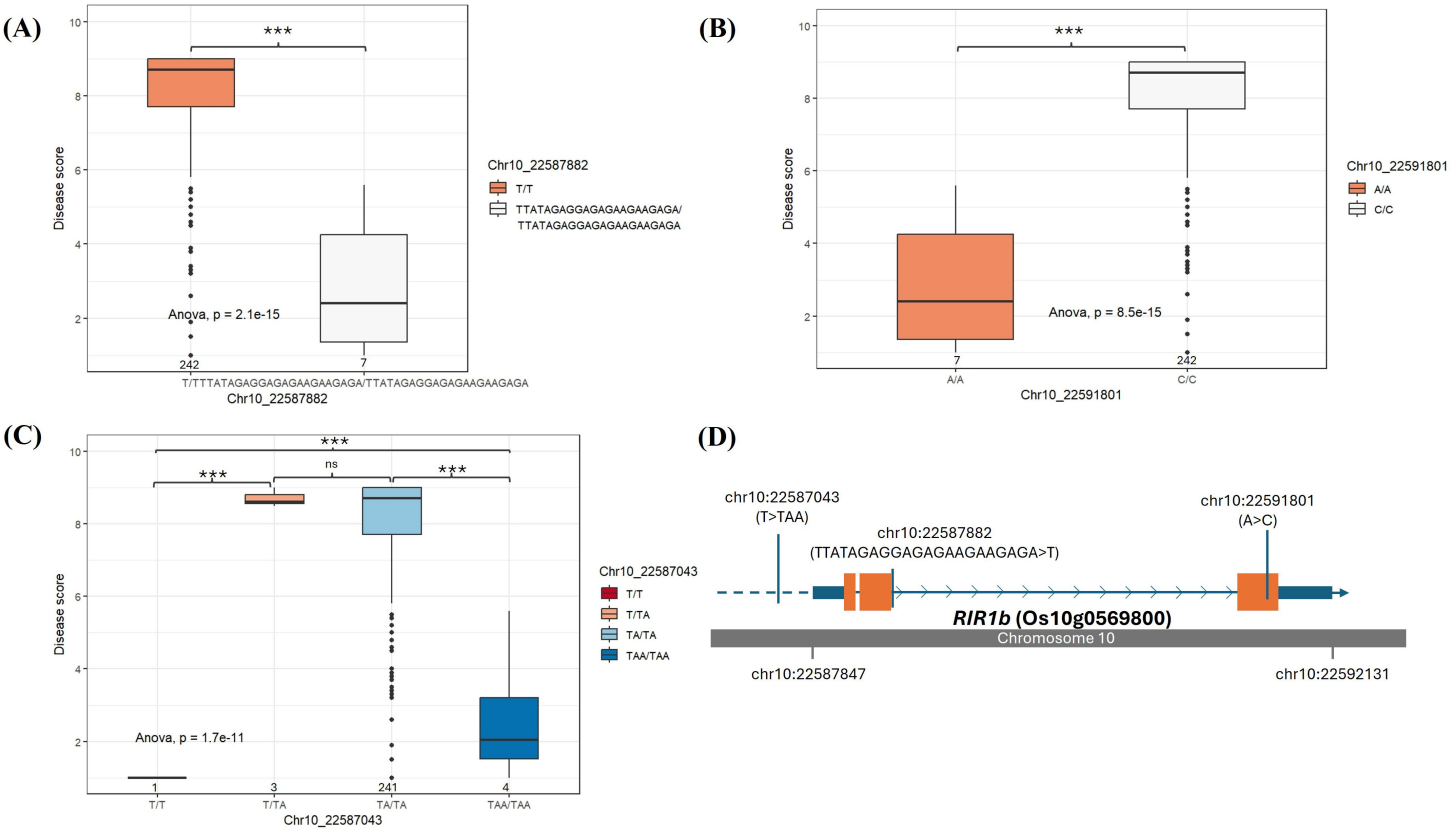
Genotype-phenotype association on *RIR1b* (Os10g0569800). The box plots display two different genotypes on **(A)** Chr10: 22587882 and **(B)** Chr10: 22591801, and **(C)** four different genotypes on Chr10:22587043 which are analyzed in rice germplasm consisting of 262 rice accessions. The median is indicated by solid horizontal lines in the box plots. **(D)** The structure of *RIR1b* (Os10g0569800) shows UTRs (small blue boxes), exons (orange boxes), introns (blue arrow lines), and upstream regulatory sequences (blue dash line). The grey bar depicts the chromosome, and the SNP positions are shown by blue vertical bars. *** p< 0.001, ns (non-significant).

Genotype-phenotype association analysis of the SNPs or InDels identified on *OsBIP1*, *POX22.3*, *OsSDF2-1*, *OsSDF2-2*, and *OsLRR-RLK2* cannot give significant separation between resistant and susceptible accessions. We could identify that the 21 bp deletion (homozygous genotype T/T) at the position of 10:22587882, which can cause a disruptive in-frame deletion in *RIR1b*, and this deletion was seen in susceptible accessions (**Figures 6A, D**, **Table 2**). Only 7 accessions with disease score of highly resistant to moderately resistant have the homozygous genotype TTATAGAGGAGAGAAGAAGAGA/TTATAGAGGAGAGAAGAAGAGA, and they also include DV85 and NDCMP49. Moreover, the same 7 accessions have a homozygous A/A genotype at the position of 10:22591801 at exon 3 of *RIR1b*, and the susceptible accessions have the homozygous C/C genotype (**Figures 6B, D**). This nucleotide change made a non-synonymous variant and amino acid change (Thr>Pro). However, according to expression data, only NDCMP49 has significant up-regulation over HCS and even DV85. From the genotype-phenotype association, there are 22 variants in the regulatory region of *RIR1b*, and 2 bp insertions at the position of 10:22587043 (TAA/TAA) were found in 4 BLS resistant genotypes, which involve NDCMP49 only (**Figures 6C, D**).

**Table 2:** List of significant variants of *RIR1b* according to genotype-phenotype association analysis.

SNP_ID	Significant codes	Multiple R^2^	Adjusted R^2^	REF	ALT	SNP effect	Impact
10_22587043	***	0.1639	0.0229	T	TA, TAA	Upstream gene variant	MODIFIER
10_22587882	***	0.4754	0.2229	TTATAGAGGAGA GAAGAAGAGA	T	Splice acceptor variant & disruptive in-frame deletion & splice region variant & intron variant	HIGH
10_22591801	***	0.4754	0.2229	A	C	Missense variant	MODERATE

***p< 0.001.

## 4 Discussion

Rice is a vital source of human nutrition, and its consumption is rising year-on-year, whilst production is constrained by stress factors, including pathogenic microorganisms (Jones and Dangl, 2006). Increases in global temperatures are expected to accelerate pathogen evolution, influence disease epidemiology, modify host-pathogen interactions, and affect vector biology, triggering the emergence of new pathogen strains and, crucially, disease outbreaks (Singh et al., 2023). It is therefore important to discover new, stable, and broad-spectrum disease-resistance genes in rice and to breed disease-resistant varieties. BLS is a bacterial disease of rice, which in the hot and humid conditions that are optimal for *Xoc*, can lead to substantial yield losses (He et al., 2012). The molecular mechanisms underlying rice resistance to BLS are still limited, as studies have focused mainly on the more important rice disease, BLB. As of now, *xa5* remains the sole major resistance gene identified for BLS resistance (Xie et al., 2014) although around 20 BLS-related QTLs have been discovered.

**Figure 7:**
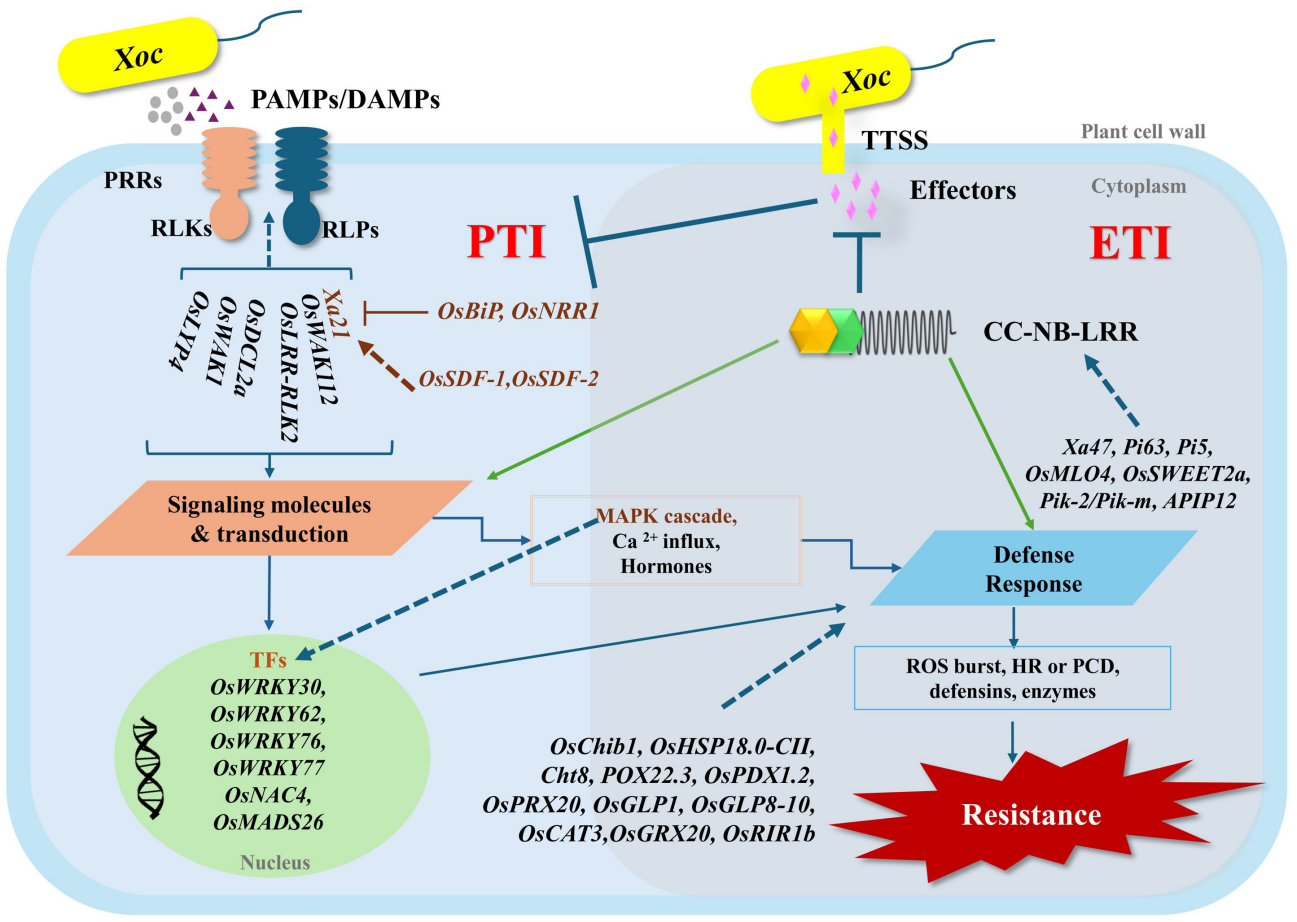
Summarized illustration of DEGs regulated in the plant defense system against *Xoc*. (PAMPs = pathogen-associated molecular patterns, DAMPs = damage-associated molecular patterns, PRRs = pattern recognition receptors, RLKs = receptor-like kinases, RLPs = receptor-like proteins, TTSS = type III secretion system, CC-NB-LRR = coiled-coil nucleotide-binding leucine-rich repeats, TFs = transcription factors, MAPK = mitogen-activated protein kinase, ROS = reactive oxygen species, HR = hypersensitive response, PTI = PAMP triggered immunity, ETI = effectors triggered immunity).

Plants use remarkably complex defense systems to resist pathogen attacks. In the first level of the plant immunity system, molecular characteristics of invading pathogens (called pathogen-associated molecular patterns/PAMPs or microbe-associated molecular patterns/MAMPs) are perceived by pattern recognition receptors (PRRs). These are receptor-like proteins or receptor-like kinases (RLPs or RLKs), and this innate immunity is defined as PAMP-triggered immunity (PTI) (**Figure 7**). The second layer of the immunity system is called effector-triggered immunity (ETI), in which the molecules secreted into host cells by pathogens are detected and elicit signals for further response through multiple defense cascades (Andersen et al., 2018). The ETI system relies on the existence of plant resistance proteins (R proteins), which frequently contain both nucleotide-binding (NB) and leucine-rich repeat (LRR) domains and are termed NLRs (nucleotide-binding leucine-rich repeats). NLRs act to recognize pathogen effectors, and this can lead to a hypersensitive response (HR) or rapid localized programmed cell death (PCD) by the plant to prevent further pathogen invasion (Nguyen et al., 2021). Both PTI and ETI recognition events can activate downstream signaling pathways containing mitogen-activated protein kinases (MAPK), calcium ion release, transcription factor activity, production of hormones and reactive oxygen species (ROS), and epigenetic regulation (Andersen et al., 2018).

In the present study, transcriptome analysis compared three different rice genotypes, which included HCS (a BLS susceptible variety), DV85 (a resistant variety with at least two resistance genes, i.e., *xa5* and *Xa7*), and NDCMP49 (a resistant variety with an unknown alternative mechanism). DV85 is well-characterized and has been used as a donor of BLS resistance in several breeding programs (Mabreja et al., 2024; Wang et al., 2005; Yasui et al., 2010). On the other hand, resistant alleles of neither the *xa5* nor the *Xa7* genes are present in NDCMP49, and an unknown resistance mechanism must be present in this variety. RNA-seq was used to assess the gene expression profiles of each genotype at 0 and 9 hpi in a series of pairwise comparisons. In paired comparisons of H9h vs H0h, D9h vs D0h, and N9h vs N0h, the HCS variety showed significantly more up- and down-regulated DEGs at 9 hpi, indicating that the successful *Xoc* infection in the susceptible interaction resulted in more changes of gene expression than the resistant interactions. This matches with other transcriptome studies against BLS. This can be usual because in susceptible interactions such as the pathogens can influence the host’s gene expression to boost their virulence and infection. Resistant plants, on the other hand, might use a more specific defense pathway, leading to smaller changes in DEGs (Lu et al., 2020; Xie et al., 2020). Between the two resistant genotypes, NDCMP49 showed more DEGs than DV85 at 9 hpi. Further analyses showed that the DEGs identified following *Xoc* infection were enriched in terms previously associated with defense mechanisms, such as ‘catalytic activity’, ‘chloroplast’ ‘mitochondrion’, ‘RNA modification’, ‘defense response’ from GO, and ‘metabolic pathways’, ‘biosynthesis of secondary metabolites’ and other pathways from KEGG (Bakade et al., 2021; Kong et al., 2020; Lu et al., 2020; Malukani et al., 2020; Matic et al., 2016; Ortiz-Morea et al., 2022; Zhou et al., 2016).

In this RNA-seq analysis, 132 different RLKs were identified, which are known to be important components of the first layer of the immunity system (Wu et al., 2016). In our analysis, only NDCMP49 showed significant up-regulation of *OsLRR-RLK2* or *OsRIR1* (*rice immunity repressor 1*) at 9 hpi, 2.4-fold in N9h vs H9h and 2.71-fold in N9h vs D9h. Interestingly, *OsRIR1* has previously been shown to suppress the defense response of rice plants to *Xoo* infection; an insertion mutant of *OsRIR1* showed increased resistance to *Xoo* infection, whilst over-expressing lines exhibited susceptibility (An et al., 2022). Its upregulation in NDCMP49 could be part of an unrecognized or indirect defense pathway unique to this variety when confronted with *Xoc*. Silencing of *OsRIR1b* affected the transcription of other genes, defense-related genes such as *OsMPK6* and *WRKY* transcription factors (Ye et al., 2020). Future investigations could study the direct interactions of *OsRIR1* with *Xoc*-specific effectors or its downstream signaling partners in NDCMP49. In the present study, the significant and high expression of *Xa21* was observed in resistant genotypes: 6.71-fold upregulation in DV85 and 2.06-fold upregulation in NDCMP49 at 9 hpi strongly implicates its role in recognizing the PAMPs emitted by *Xoc*. *Xa21* is a broad-spectrum resistance gene first identified in the wild rice species *Oryza longistaminata.* There have been many studies of *Xa21*-mediated immunity, and its signaling pathways are well-characterized. The *Xa21* (*RLK*) recognizes the pathogen ligand and activates host intracellular defense responses (Song et al., 1995) including downstream cascades key proteins such as mitogen-activated protein kinase 5 (*MAPK5*) and transcription factors (*OsWRKY62* and *OsWRKY76*) (Peng et al., 2008; Seo et al., 2011). The significant induction of *Xa21* suggests a rapid and effective PAMP event in resistant genotypes, leading to an instant defense response. This broad-spectrum activity of *Xa21* makes it a valuable target for enhancing resistance against diverse *Xanthomonas* strains. Wall-associated receptor-like kinases (WAKs) are a distinct class of RLK that can integrate cell wall integrity with defense signaling (de Oliveira et al., 2014). The upregulation of *OsWAK1* in H9h vs H0h and N9h vs N0h indicates its general role in early *Xoc* detection, possibly in response to cell wall perturbations induced not only by bacteria but also by fungal activities like rice blast (Li et al., 2009). High upregulation of *OsWAK112d* only in the resistant genotype N9h suggests an alternative role in response to bacterial infection, because it is known to have a negative regulatory role in salt stress and rice blast resistance (Delteil et al., 2016; Lin et al., 2021). Therefore, its high regulation in response to *Xoc* suggests a novel, possibly unique, resistance mechanism in NDCMP49. Additionally, our data also revealed that several WAK genes (such as *OsWAK2*, *OsWAK5*, *OsWAK16*, *OsWAK79*, *OsWAK80*, *OsWAK97*, etc) are regulated differentially, and this implies a specialized, non-redundant function of each in the overall defense against *Xoc*, warranting further functional annotation. The significant upregulation of one of the Dicer-like Enzymes, *OsDCL2a* (11.28-fold in D9h vs H9h and 9.91-fold in N9h vs H9h) was observed only in resistant genotypes compared to HCS and also no regulation was detected in H9h vs H0h. It is an interesting observation, especially when compared to the contrasted finding with its downregulation in response to viral infection to suppress RNAi (Pumplin and Voinnet, 2013; Wang et al., 2021; Xu and Zhou, 2017). This suggests a potentially positive role of *OsDCL2a* in the rice defense system against *Xoc*. While DCLs are primarily known for their role in RNA silencing, one possible explanation of their involvement in defense against *Xoc* is that DCLs process bacterial small RNAs or activate the host’s gene expression through microRNA pathways that facilitate plant defense. This finding could be a force to explore the new aspects of RNA-mediated immunity of plants in response to bacterial pathogens. Dissecting the role of these DEGs related to PTI plant immunity system and cross-talk or their redundant contributions to BLS resistance would be a major challenge.

Additionally, the transcriptome analysis revealed that several categories of transcription factors, predominantly, members of *WRKY*, *NAC*, *MYB*, and *bHLH* families, were differentially expressed following *Xoc* infection. Among WRKY transcription factors, *OsWRKY77* showed significant upregulation in the resistant genotype NDCMP49 at 9 hpi compared to H9h and D9h with fold change values of 5.23 and 7.25, respectively. Over-expression of *OsWRKY77* in *Arabidopsis thaliana* has been shown to boost resistance to *Pseudomonas syringae* pv. *tomato* by increasing defense-related genes such as *PR1*, *PR2*, and *PR5* (Lan et al., 2013). This suggests that *OsWRKY77* is a promising candidate for conferring basal resistance against *Xoc* in rice. Several members of the OsWRKY IIa subfamily, such as *OsWRKY62* and *OsWRKY76*, are known to play a role in the *Xa21*-mediated resistance pathway against *Xoo*. While *OsWRKY62* suppresses defense when overexpressed (Peng et al., 2008), knocking down either *OsWRKY62* or *OsWRKY76* enhances resistance to *Xoo* (Peng et al., 2010). Interestingly, *OsWRKY62* also regulates resistance to rice blast and BLB by activating phytoalexin production (Fukushima et al., 2016). In our study, both *OsWRKY62* and *OsWRKY76* showed significantly higher expression; *OsWRKY62* was 4.2- and 7.4-fold upregulated in D9h and N9h, respectively, and *OsWRKY76* was 4.8- and 8.0-fold upregulated in D9h and N9h, respectively, than H9h. This strong indication suggests that these two transcription factors are likely to significantly enhance the basal resistance against *Xoc*, and their influence on additional compounds, including phytoalexins and other defense-related genes, merits deeper exploration. *OsWRKY30* is also a known positive regulator of basal resistance and salicylic acid (SA) signaling during *Xoo* infection (Han et al., 2013). In our results, D9h showed approximately 5-fold and 4.25-fold higher upregulation of *OsWRKY30* than N9h and H9h, respectively. Given that HR is often linked to increased levels of SA, it would be valuable to investigate if this high expression of *OsWRKY30* correlates with increased SA levels in DV85 and whether this differs in NDCMP49. *OsNAC4* is a well-studied member of the NAC family in rice, and it was shown to be involved in HR because its overexpression causes cell death, and knockdown reduces cell death (Kaneda et al., 2009). In our results, *OsNAC4* expression was regulated only in DV85, but surprisingly, it was down-regulated by approximately -2.35-fold at D9h. This suggests that DV85 might utilize alternative, *OsNAC4*-independent pathways, for instance, the *xa5*-mediated pathway, highlighting the complexity and diversity of resistance mechanisms in rice. Therefore, future studies focusing on functional studies, for example, overexpression or knocking down of *OsNAC4* in DV85, especially under *Xoc*-infected conditions, and investigation of any regulatory link between *OsNAC4* and *xa5* should be followed.

In our study, we also identified DEGs related to the ETI system, most of which belong to the NLR domain-containing protein family. These proteins, encoded by R genes, recognize pathogen proteins and activate signaling to trigger a plant immune response (Bezerra-Neto et al., 2019). Fungal disease resistance genes for *Magnaporthe oryzae* such as *Pi63*, *Pik2*, and *Pik2*-like genes (Lee et al., 2009; Xu et al., 2014; Zhai et al., 2011) are highly upregulated in NDCMP49. The fact that *Xoc* infection induces the expression of these anti-fungal R genes raises a question: whether bacterial and fungal effectors share the structural and functional similarities to be recognized by these proteins or shared signaling pathways and hormonal crosstalk are activated during *Xoc* infection. Similarly, R gene *Xa47* was highly upregulated only in NDCMP49 providing strong evidence for distinct mechanisms between DV85 and NDCMP49. In *Xoo*-rice pathosystem *Xa47* is disrupted by *Xoo* TALE (Lu et al., 2022). It suggests that *Xoc* might possess TALE or TALE-like effectors that can recognize *Xa47* in NDCMP49 or DV85 does not have a functional *Xa47* allele. Furthermore, significant downregulation of *SWEET* genes such as *OsSWEET1a*, *OsSWEET2a*, and *OsSWEET3a* were observed in resistant genotypes at 9hpi. Pathogens have evolved mechanisms to hijack these sugar transporters, thereby securing the nutrient resources necessary for the successful progression of infection, and therefore, *SWEET* genes may act as susceptibility factors during bacterial and fungal pathogen interactions (Gao et al., 2021; Gupta, 2020). However, it is interesting to see the downregulation of *OsSWEET1a* and *OsSWEET2b*, even in the susceptible genotype at 9 hpi with fold change values of -3.65 and -10.47, respectively. This finding differs from previous studies where *Xoo* utilizes TALEs to upregulate specific host SWEET genes (e.g., *OsSWEET11*/*Xa13* and *OsSWEET13*/*Xa41*) to facilitate pathogen nutrition (Antony et al., 2010; Streubel et al., 2013; Yu et al., 2011). Our results suggest that the *Xoc* strain (1NY2-2) used in this study may not possess TAL effectors that can target activation of *OsSWEET1a* and *OsSWEET2b*. This result is further supported by upregulation of susceptibility gene *OsMLO4* in NDCMP49. Although *Xanthomonas* species are characterized by their effectors, variation of effectors can be observed between different strains from different geographic areas and genetic backgrounds (Antony et al., 2010; Streubel et al., 2013; Yu et al., 2011). All together, we therefore propose further investigations, such as analyzing the effector profile of 1NY2-2 and the disruption of these susceptibility genes, to reduce the compatibility between the rice host and *Xoc* pathogen towards the enhancement of broad-spectrum resistance mechanisms and thereby elevate BLS disease resistance.

The *Xa21-RLK* gene was highly up-regulated in the two resistant varieties, as outlined above, and this suggests that the well-characterized downstream components of the *Xa21* pathway might also be critical for rice’s defense against *Xoc*. For example, negative regulator Bip3 which exerts a negative effect on immunity by reducing *Xa21* stability (Park et al., 2010), was up-regulated 3.22-fold higher in N9h than H9h. The *Negative Regulator of Resistance 1* (*OsNRR1*) gene affects *Xa21*-mediated resistance, basal resistance, and age-related resistance, and its overexpression leads to increased susceptibility to *Xoo* infection (Chern et al., 2005). In our experiment, *OsNRR1* was highly downregulated with a 7-fold decrease at 9 hpi in the susceptible variety, HCS, but in contrast was up-regulated in both resistant genotypes. This contrasting expression of *OsNRR1* particularly shows the complex defense mechanism of rice’s response to pathogens. Downregulation of *OsNRR1* in the susceptible genotype aligns with its negative regulatory role. Alternatively, the regulation of *OsNRR1* may be transiently induced in resistant genotypes, and therefore, further time-course analysis would be beneficial. However, upregulation in resistant genotypes might suggest that *OsNRR1’s* precise function or its downstream interactions might differ in the context of *Xanthomonas* species. *Xa21* binding protein 25 (*XB25*, previously known as *OsBIANK1*), which belongs to the plant-specific ankyrin-repeat (PANK) family, is required to maintain resistance to *Xoo*. *XB25* downregulation results in decreased levels of *Xa21* and represses resistance (Jiang et al., 2013). This gene was upregulated in all three genotypes at 9 hpi, in our dataset, suggesting that it is not only essential for the stability or proper functioning of the *Xa21* complex but also important as an early defense for basal resistance. Other genes crucial for *Xa21*-mediated immunity, such as stromal cell-derived factors *OsSDF2-1* and *OsSDF2-2* (Park et al., 2013), were also upregulated in N9h but not in D9h. These distinct *Xa21*-mediated defense strategies between NDCMP49 and DV85 indicate that *Xa21*-mediated signaling plays a role in resistance to *Xoc* infection, probably genotype-dependent, in addition to its well-documented role in combating *Xoo* infection.

Not only the ETI system, but our current study also revealed the contribution of defense related genes especially in NDCMP49. For example, one of heat shock proteins, *OsHsp18.0* was strongly up-regulated in N9h, with 6.24- and 7.35-fold up-regulation compared to H9h and D9h, respectively. *OsHsp18.0* overexpression increases resistance to BLB in an *Xoo* susceptible variety, whereas silencing reduces resistance (Kuang et al., 2017; Sarkar et al., 2009). In our experiment, given its known positive role in *Xoo* resistance, it likely contributes to a robust defense against *Xoc* as well, potentially via similar mechanisms. Although heat shock proteins are linked to general stress, this highly specific upregulation of *OsHsp18.0* in NDCMP49 provides a clue to its active contribution to disease resistance beyond general stress response. Moreover, upon *Xoc* infection, pathogenesis-related rice chitinases *Oschib1* and *Cht8* showed significant and high upregulation in NDCMP49. As rice chitinase *Oschib1* has been shown to inhibit the growth of pathogenic fungi *Fusarium solani* and have antifungal activity (Tanaka et al., 2023), our finding suggests a broad activation of PR genes to counteract diverse potential threats. In addition, our study also highlights the crucial role of stomatal immunity against *Xoc* infection because both resistant genotypes show upregulation of a pyridoxal phosphate synthase gene involved in vitamin B6 synthesis, *OsPDX1.2*, at 9 hpi. This finding is particularly important because *Xoc* is known to overcome stomatal immunity by its effector protein, *AvrRxo1*. Targeting and manipulation of *OsPDX1.2* by *Xoc* effector protein hinders the stomatal immunity and lowers vitamin B6 levels (Liu et al., 2022). These combined observations strongly suggest that stomatal immunity, supported by the enhanced activity of *OsPDX1.2*, plays a vital role in the resistance of both DV85 and NDCMP49 against *Xoc*. Perhaps unsurprisingly, our work implicated the regulation of antioxidants such as peroxidases (*OsPOX22* and *OsPrx30*) and glutaredoxin (*OsGRX20*) and they are highly upregulated in both resistant genotypes and *OsPOX22* exhibited higher expression in N9h than D9h. However, the expression of catalase (*OsCAT3*) shows subtle downregulation only in N9h vs D9h. The onset of a pathogen attack induces the accumulation of reactive oxygen species (ROS), precipitating an oxidative burst that functions as an early defense mechanism by signaling the HR. Although low concentrations of ROS are valuable signals, high ROS concentrations can be detrimental and therefore, plants rely on these ROS scavenging enzymes (Akbar et al., 2023). These findings basically highlight the sophisticated overview of the plant antioxidant system. Further research is needed to elucidate how these antioxidants might integrate with other plant defense pathways, including hormone signaling (like SA and JA) and other PR genes, following *Xoc* infection. Moreover, with the availability of CRISPR/Cas9 technology and other biotechnologies, these antioxidant genes can be used as breeding targets not only for stress resistance but also for rice yield improvement with enhanced adaptability to changing climatic conditions.

Rapid alkalinization factors (RALFs) are a distinct family of small, secreted plant peptides that have diverse roles in plants’ various biological processes (Sharma et al., 2016). In chickpea, infection by the *Fusarium oxysporum* f. sp. *Ciceri* (Race 1) induced higher *RALF* expression in resistant plants compared to susceptible ones. *RALF* production in chickpea, in response to wounding and stress, was proposed to increase pH, which is detrimental to the fungus, and *RALF* peptides might also act as decoys for plant R proteins (Gupta et al., 2010a, 2010b). In *Arabidopsis*, nematode and drought stress led to stronger induction of *RALF8*, *RALF23*, *RALF33*, and *RALF34* than either stress alone. However, overexpression of *RALF8* in *Arabidopsis* surprisingly caused significant intolerance to both drought and nematode infection (Atkinson et al., 2013). In rice, although studies are still limited, *OsRALF26* is induced by *Xoo* infection during *Xa21*-mediated immunity and plays a novel role in strengthening this immune response. When it is overexpressed, *OsRALF26* triggers various immune responses, including defense gene induction and ROS production, leading to enhanced resistance against *Xoo* in rice (Kwon et al., 2024). Similarly, overexpression of *OsRALF30*/*RIR1b*, enhances resistance to rice blast disease (Schaffrath et al., 2000). In our experiment, *OsRALF30*/*RIR1b* was very highly downregulated in DV85, with a D9h vs H9h FC value of -8.15. In contrast, *RIR1b* was highly upregulated in N9h (N9h vs D9h with fold change of 14.52), suggesting a positive role of this alkalization factor gene in the NDCMP49 regarding response to *Xoc* infection. According to allele mining performed by using whole-genome re-sequencing data of our rice germplasm, the genotype-phenotype association analysis revealed that, in *RIR1b*, a 21 bp deletion (homozygous genotype T/T) at the position of 10:22587882, causing a disruptive in-frame deletion and 1 nucleotide change (A>C) at 10:22591801 of exon 3 makes most of the accessions susceptible. Although there are 22 variants upstream of *RIR1b*, only 2 bp insertions at the position of 10:22587043 (TAA/TAA) explained the significant up-regulation of *RIR1b* in NDCMP49 over DV85 and HCS. This finding indeed points out that *RIR1b* should be considered as one of the future breeding targets that could deliver broad-spectrum resistance to rice diseases. Investigating the link between *RIR1b* and its downstream pathways, such as ROS production, induction of PR genes, or hormonal cascades, would define its precise mechanism in *Xoc* resistance.

Taken together, this study reveals that inoculation with *Xoc* elicits widespread differential gene expression, with the susceptible genotype exhibiting a substantially higher number of DEGs compared to the resistant genotypes. Among these DEGs, critical regulators of plant defense, such as receptor-like kinases, NBS-LRR proteins, and several transcription factors, central to both PTI and ETI, were actively modulated. One key challenge is how to continue further with these DEGs effectively because transcriptome study only delivers the expression information of what genes are turned on/off but not their functional changes. However, this present study combined with analysis of sequence variation of selected DEGs in broader germplasm may give a hint to work further such as functional exploration using integrative multi-omics approaches, potentially reinforcing immunity against *Xoc* infection. Moreover, studies on post-transcriptional and post-translational events of identified DEGs, editing of susceptibility genes like SWEETs, identification of effector profiles of local *Xoc* strains and their targets in hosts’ promoter and pyramiding to stack these genes via breeding programs would advance achieving of broad-spectrum disease resistance. Collectively, these insights offer a powerful framework for advancing the development of BLS-resistant rice genotypes, laying the groundwork for more effective and sustainable resistance breeding strategies.

## 5 Conclusion

In summary, our results reveal new components that are likely to be involved in resistance to *Xoc*. These include components previously implicated in responses to infection by the closely related bacterial pathogen *Xoo* and fungal pathogens. Perhaps more interestingly, we identify several genes that are differentially regulated in the NDCMP49 rice cultivar in comparison to other varieties, as these may reveal clues to the molecular underpinnings of its alternative resistance mechanism. Of these genes, one PR protein, *RIR1b*, shows significant expression in NDCMP49 than the rest of the genotypes. Several genes encoding components previously associated with resistance to *Xoo* via an *Xa21*-based response were upregulated in both resistant varieties. Thus, we propose that, in addition to the well-characterized *xa5*-mediated response to *Xoc*, at least some cultivars of rice can utilize alternative mechanisms. The identification of these new components in the resistance response to *Xoc* should help breeders and molecular biologists to create new crop varieties that can withstand BLS and perhaps other diseases.

## Data Availability

The data presented in the study are deposited in the NCBI repository, accession number PRJNA1256609.

## Supplementary material

Supplementary Figure 1 Morphological tests of 1NY2-2. **(A)** Colony appearance of 1NY2-2 isolate on Nutrient Agar media (3 g of beef extract, 5 g of peptone and 15 g agar per liter of media) after incubation at 30°C for 3-4 days. **(B)** Appearance of gram negative 1NY2-2 under microscope. (To gram stain 1NY2-2, fresh culture of 24-48 hour-aged bacteria were spread in 1-2 drops of clean water on glass slide. To make bacterial smear, bacteria cells were then heat fixed and were stained with 1-2 drops of crystal violet for 1 min. Then washed with sterilized distilled water and Gram’s iodine solution was poured into it. Then it was washed with 95% ethanol, water and blotted dry. Finally, they were counter stained with Safranin O for 30 seconds, rinsed with water and left to dry. The bacterial cells were observed under microscope and gram-negative bacteria were seen pink to red).

Supplementary Figure 2 GO enrichment analysis of DEGs of all contrasting genotypes at 9 hpi compared to 0 hpi. **(A)** GO categories that are significantly enriched in HCS. **(B)** GO categories which are enriched in DV85. **(C)** GO terms enriched in NDCMP49. (The x-axis illustrates the number of DEGs in each GO term and the y-axis describes the enriched GO categories. MF= Molecular Function, CC= Cellular Component, BP= Biological Process).

Supplementary Figure 3 S1. GO enrichment analysis of DEGs of all contrasting genotypes at 9 hpi compared to 0 hpi. **(A)** GO categories that are significantly enriched in HCS. **(B)** GO categories which are enriched in DV85. **(C)** GO terms enriched in NDCMP49. (The x-axis illustrates the number of DEGs in each GO term and the y-axis describes the enriched GO categories. MF= Molecular Function, CC= Cellular Component, BP= Biological Process).

Supplementary Figure 4 Hierarchical clustering of DEGs, which are important in pathogen detection, signal transduction, and defense response, of all genotypes at 0 hpi and 9 hpi. **(A)** Heatmap showing the expression profiles of receptor-like kinases (RLKs), which play crucial roles as pattern recognition receptors (PRR) in plant immunity. **(B)** Heatmap showing the expression profiles of transcription factors (TFs) in which the majority are involved in transcription of downstream genes related with defense response to pathogen and send signals for downstream pathways. **(C)** Heatmap showing the expression pattern of other important proteins that have essential roles in signal transduction to initiate defense response and resistance. (Data corresponds to Trimmed Mean of M value (TMM). Z-scores and color key represent the scaled expression values, with green for low expression and red for high expression. Clustering of genes is shown on the left side of the figure and gene names are shown on the right. Repeated gene names are indications of transcript variants).

Supplementary Figure 5 Validation of seven by qRT-PCR. DEGs were randomly selected from transcriptome analysis, and primers were designed for qRT-PCR validation. Rice actin was used as an internal control. The bar graphs represent the relative expression value from qRT-PCR and TMM transcript counts from RNA-seq data.
