# Supplementary figures and images for: Transcriptomic analysis of rice cultivars with distinct resistance mechanisms to Xanthomonas oryzae pv. oryzicola reveals novel components and candidate genes associated with bacterial leaf streak
Source: Front Plant Sci. 2025 Sep 9;16:1613802. doi: 10.3389/fpls.2025.1613802 (PMC12459299; doi:10.3389/fpls.2025.1613802)

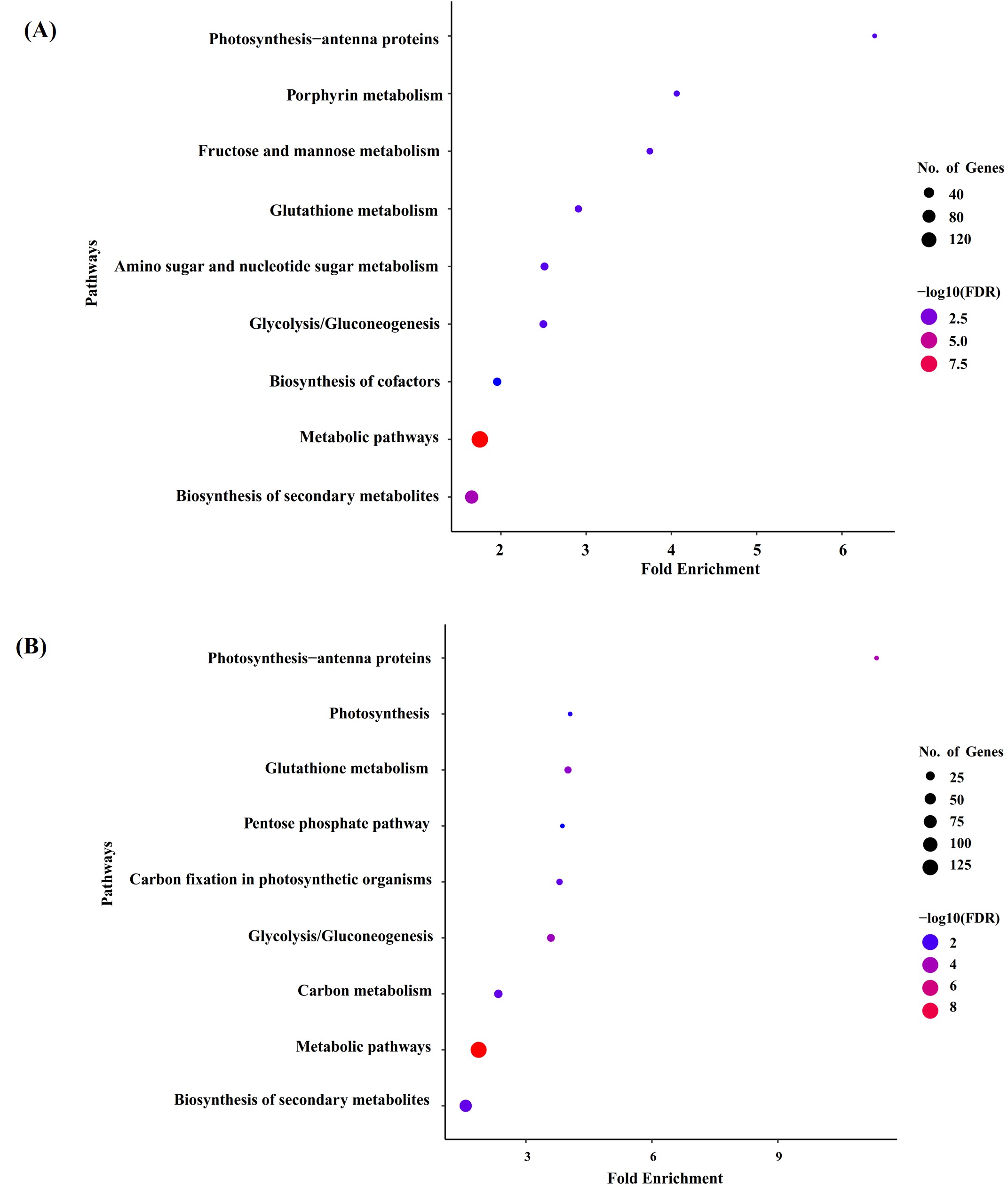

Supplement: Supplementary Figure 1 — Morphological tests of 1NY2-2. (A) Colony appearance of 1NY2-2 isolate on Nutrient Agar media (3 g of beef extract, 5 g of peptone and 15 g agar per liter of media) after incubation at 30°C for 3-4 days. (B) Appearance of gram negative 1NY2-2 under microscope. (To gram stain 1NY2-2, fresh culture of 24-48 hour-aged bacteria were spread in 1-2 drops of clean water on glass slide. To make bacterial smear, bacteria cells were then heat fixed and were stained with 1-2 drops of crystal violet for 1 min. Then washed with sterilized distilled water and Gram’s iodine solution was poured into it. Then it was washed with 95% ethanol, water and blotted dry. Finally, they were counter stained with Safranin O for 30 seconds, rinsed with water and left to dry. The bacterial cells were observed under microscope and gram-negative bacteria were seen pink to red). [file DataSheet1.zip › Supplementary/Figure S3.jpg]

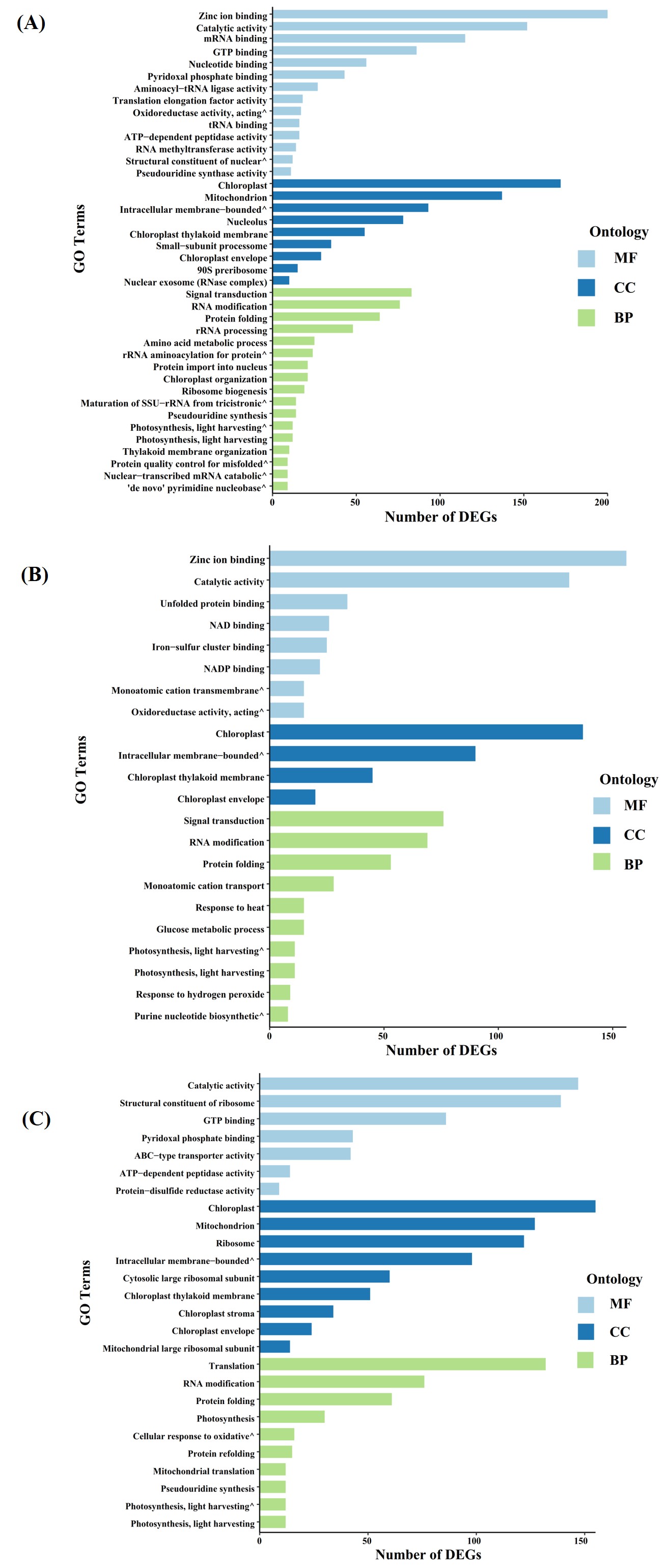

Supplement: Supplementary Figure 1 — Morphological tests of 1NY2-2. (A) Colony appearance of 1NY2-2 isolate on Nutrient Agar media (3 g of beef extract, 5 g of peptone and 15 g agar per liter of media) after incubation at 30°C for 3-4 days. (B) Appearance of gram negative 1NY2-2 under microscope. (To gram stain 1NY2-2, fresh culture of 24-48 hour-aged bacteria were spread in 1-2 drops of clean water on glass slide. To make bacterial smear, bacteria cells were then heat fixed and were stained with 1-2 drops of crystal violet for 1 min. Then washed with sterilized distilled water and Gram’s iodine solution was poured into it. Then it was washed with 95% ethanol, water and blotted dry. Finally, they were counter stained with Safranin O for 30 seconds, rinsed with water and left to dry. The bacterial cells were observed under microscope and gram-negative bacteria were seen pink to red). [file DataSheet1.zip › Supplementary/Figure S2.jpg]

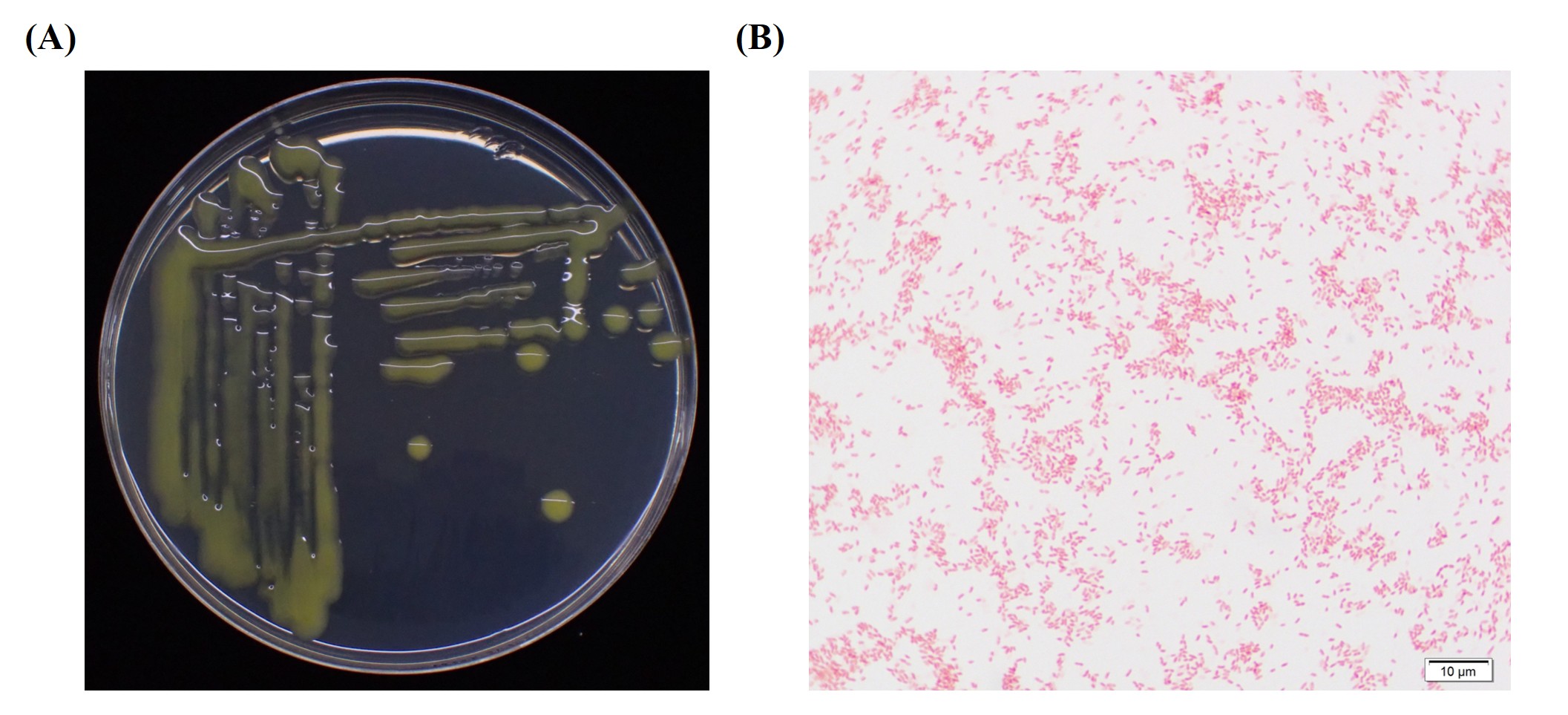

Supplement: Supplementary Figure 1 — Morphological tests of 1NY2-2. (A) Colony appearance of 1NY2-2 isolate on Nutrient Agar media (3 g of beef extract, 5 g of peptone and 15 g agar per liter of media) after incubation at 30°C for 3-4 days. (B) Appearance of gram negative 1NY2-2 under microscope. (To gram stain 1NY2-2, fresh culture of 24-48 hour-aged bacteria were spread in 1-2 drops of clean water on glass slide. To make bacterial smear, bacteria cells were then heat fixed and were stained with 1-2 drops of crystal violet for 1 min. Then washed with sterilized distilled water and Gram’s iodine solution was poured into it. Then it was washed with 95% ethanol, water and blotted dry. Finally, they were counter stained with Safranin O for 30 seconds, rinsed with water and left to dry. The bacterial cells were observed under microscope and gram-negative bacteria were seen pink to red). [file DataSheet1.zip › Supplementary/Figure S1.jpg]

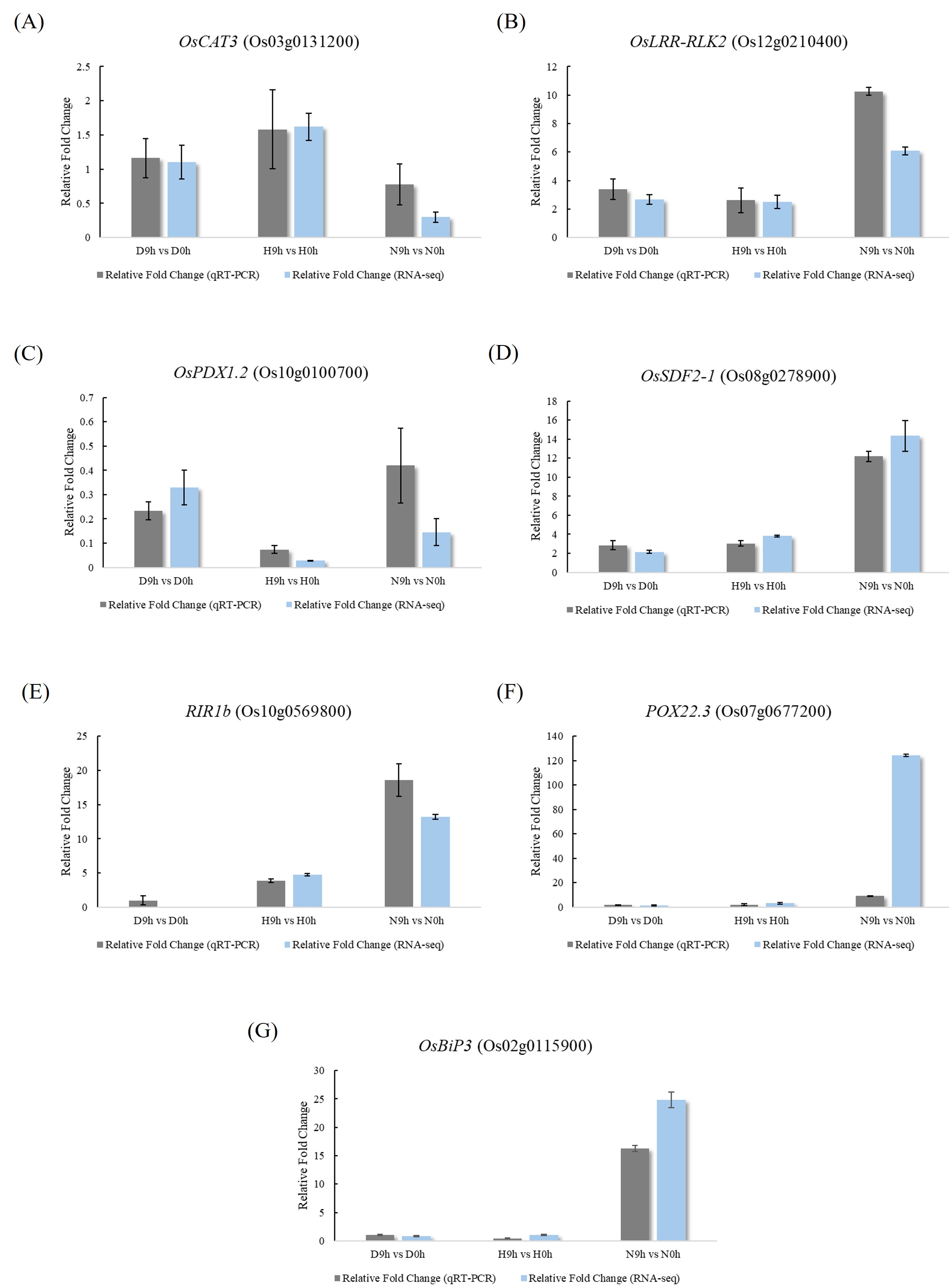

Supplement: Supplementary Figure 1 — Morphological tests of 1NY2-2. (A) Colony appearance of 1NY2-2 isolate on Nutrient Agar media (3 g of beef extract, 5 g of peptone and 15 g agar per liter of media) after incubation at 30°C for 3-4 days. (B) Appearance of gram negative 1NY2-2 under microscope. (To gram stain 1NY2-2, fresh culture of 24-48 hour-aged bacteria were spread in 1-2 drops of clean water on glass slide. To make bacterial smear, bacteria cells were then heat fixed and were stained with 1-2 drops of crystal violet for 1 min. Then washed with sterilized distilled water and Gram’s iodine solution was poured into it. Then it was washed with 95% ethanol, water and blotted dry. Finally, they were counter stained with Safranin O for 30 seconds, rinsed with water and left to dry. The bacterial cells were observed under microscope and gram-negative bacteria were seen pink to red). [file DataSheet1.zip › Supplementary/Figure S5.jpg]

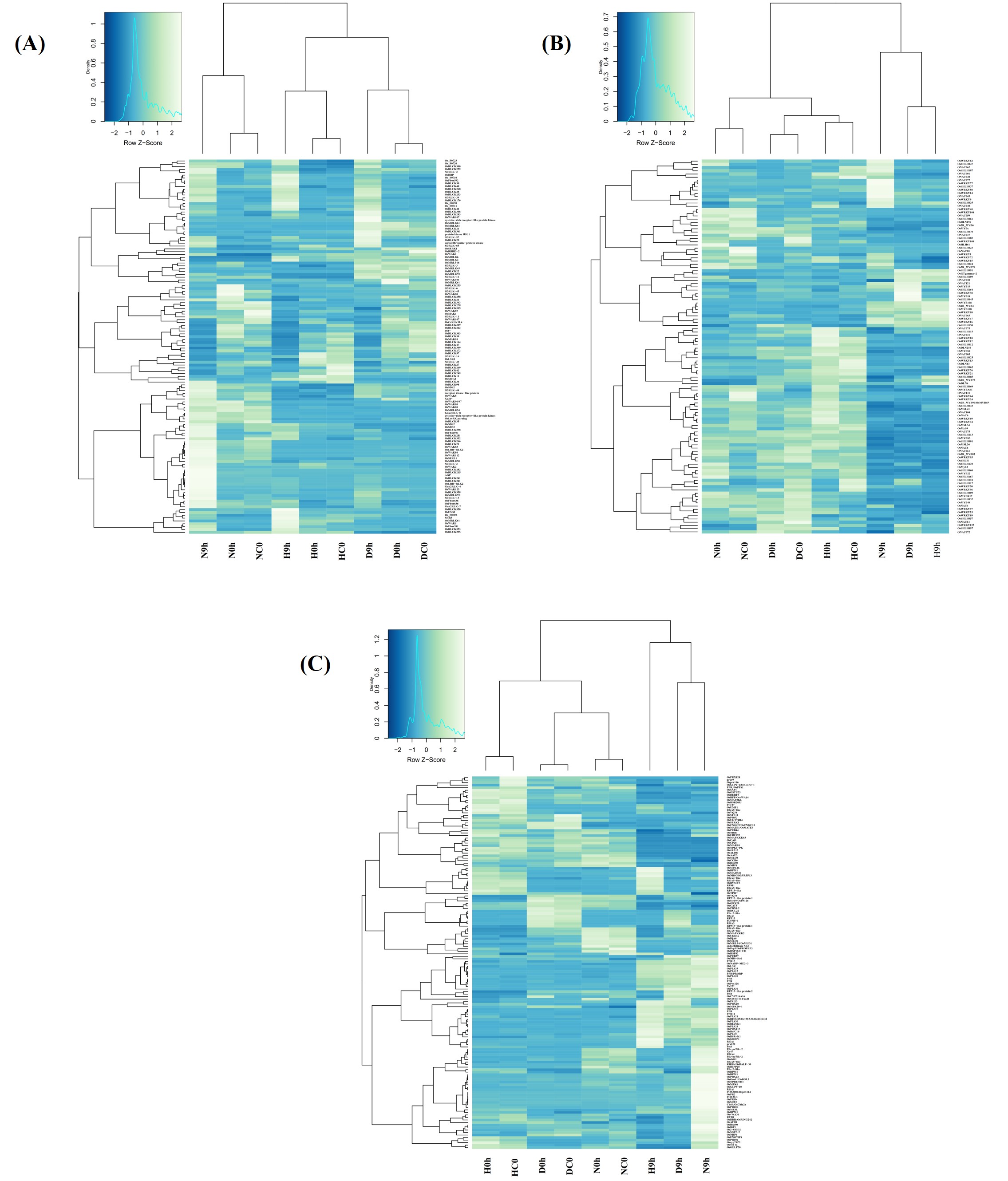

Supplement: Supplementary Figure 1 — Morphological tests of 1NY2-2. (A) Colony appearance of 1NY2-2 isolate on Nutrient Agar media (3 g of beef extract, 5 g of peptone and 15 g agar per liter of media) after incubation at 30°C for 3-4 days. (B) Appearance of gram negative 1NY2-2 under microscope. (To gram stain 1NY2-2, fresh culture of 24-48 hour-aged bacteria were spread in 1-2 drops of clean water on glass slide. To make bacterial smear, bacteria cells were then heat fixed and were stained with 1-2 drops of crystal violet for 1 min. Then washed with sterilized distilled water and Gram’s iodine solution was poured into it. Then it was washed with 95% ethanol, water and blotted dry. Finally, they were counter stained with Safranin O for 30 seconds, rinsed with water and left to dry. The bacterial cells were observed under microscope and gram-negative bacteria were seen pink to red). [file DataSheet1.zip › Supplementary/Figure S4.jpg]
